# A newly characterized malaria antigen on erythrocyte and merozoite surfaces induces parasite inhibitory antibodies

**DOI:** 10.1084/jem.20200170

**Published:** 2021-08-03

**Authors:** Ian C. Michelow, Sangshin Park, Shu-Whei Tsai, Bonnie Rayta, Charisse Flerida A. Pasaje, Sara Nelson, Angela M. Early, Anne P. Frosch, George Ayodo, Dipak K. Raj, Christina E. Nixon, Christian P. Nixon, Sunthorn Pond-Tor, Jennifer F. Friedman, Michal Fried, Patrick E. Duffy, Karine G. Le Roch, Jacquin C. Niles, Jonathan D. Kurtis

**Affiliations:** 1 Department of Pediatrics, Division of Infectious Diseases, The Warren Alpert Medical School of Brown University, Providence, RI; 2 Center for International Health Research, Rhode Island Hospital, Providence, RI; 3 Graduate School of Urban Public Health & Department of Urban Big Data Convergence, University of Seoul, Seoul, Republic of Korea; 4 Department of Biological Engineering, Massachusetts Institute of Technology, Cambridge, MA; 5 Infectious Disease and Microbiome Program, Broad Institute of Massachusetts Institute of Technology and Harvard, Cambridge, MA; 6 Department of Medicine, Hennepin Healthcare Research Institute, University of Minnesota, Minneapolis, MN; 7 Kenya Medical Research Institute, Centre of Global Health Research, Kisumu, Kenya; 8 Jaramogi Oginga Odinga University of Science and Technology, Bondo, Kenya; 9 Department of Pathology and Laboratory Medicine, The Warren Alpert Medical School of Brown University, Providence, RI; 10 Department of Pediatrics, The Warren Alpert Medical School of Brown University, Providence, RI; 11 Laboratory of Malaria Immunology and Vaccinology, National Institute of Allergy and Infectious Diseases, National Institutes of Health, Rockville, MD; 12 Department of Molecular, Cell and Systems Biology, Center for Infectious Disease and Vector Research, University of California, Riverside, Riverside, CA

## Abstract

We previously identified a *Plasmodium falciparum* (*Pf*) protein of unknown function encoded by a single-copy gene, *PF3D7_1134300*, as a target of antibodies in plasma of Tanzanian children in a whole-proteome differential screen. Here we characterize this protein as a blood-stage antigen that localizes to the surface membranes of both parasitized erythrocytes and merozoites, hence its designation as *Pf* erythrocyte membrane and merozoite antigen 1 (*Pf*EMMA1). Mouse anti-*Pf*EMMA1 antisera and affinity-purified human anti-*Pf*EMMA1 antibodies inhibited growth of *P. falciparum* strains by up to 68% in growth inhibition assays. Following challenge with uniformly fatal *Plasmodium berghei *(*Pb*) ANKA, up to 40% of mice immunized with recombinant *Pb*EMMA1 self-cured, and median survival of lethally infected mice was up to 2.6-fold longer than controls (21 vs. 8 d, P = 0.005). Furthermore, high levels of naturally acquired human anti-*Pf*EMMA1 antibodies were associated with a 46% decrease in parasitemia over 2.5 yr of follow-up of Tanzanian children. Together, these findings suggest that antibodies to *Pf*EMMA1 mediate protection against malaria.

## Teaser

*Plasmodium falciparum* erythrocyte membrane and merozoite antigen 1 (*Pf*EMMA1) is a newly characterized malaria protein expressed on the surface of parasitized erythrocytes and merozoites that induces protective antibodies in functional assays, a human epidemiological study, and a mouse immunization model.

## Introduction

The human malaria parasite, *Plasmodium falciparum* (*Pf*), claims >400,000 lives each year despite decades of intensive public health interventions in endemic areas (WHO, 2020). Global efforts to combat malaria have met with rapidly emerging resistance to frontline antimalarial agents and insecticides (Hemingway et al., 2016). In addition, RTS,S, the most advanced malaria vaccine candidate, had limited efficacy and durability in phase III trials (Olotu et al., 2016). At least 50 predominantly subunit malaria vaccines as well as whole sporozoite vaccines are currently under investigation in preclinical or clinical trials (WHO, 2017). However, the subunit candidates are derived from <25 unique antigens. The organism’s complex biology, poorly defined protective immunity, capacity to evade immune detection, and extensive genetic variability represent major challenges to discover effective immunogens and immunotherapeutic strategies.

There is an emerging consensus that next-generation subunit vaccines will need to combine multiple conserved antigens from different life cycle stages to achieve and sustain highly effective strain-transcending, sterilizing, and transmission-blocking immunity against malaria (Draper et al., 2015; Ewer et al., 2015; Osier et al., 2014). In particular, asexual blood-stage components must induce antibodies that (1) confer highly effective clinical protection by inhibiting the capacity of parasite-infected RBCs (iRBCs) to cytoadhere, sequester, or acquire nutrients; (2) counter immune evasion; and/or (3) prevent parasite invasion or egress from RBCs (Douglas et al., 2011; Miura, 2016; Nilsson Bark et al., 2018).

We previously reported results from a whole-proteome differential screen of a blood-stage *Pf*3D7 strain cDNA Lambda Zap library (MR4) using plasma from malaria-resistant or -susceptible 2-yr-old Tanzanian children enrolled in a longitudinal birth cohort (Raj et al., 2014). We identified PF3D7_1134300, a putative protein encoded by a gene on chromosome 11, as a target of antibodies in plasma from resistant but not susceptible Tanzanian children. Bioinformatics analysis of this protein predicts a 6,684-bp single-copy gene that has syntenic orthologues in all human (*Pf*, *Plasmodium vivax*, *Plasmodium ovale*, *Plasmodium malariae*, and *Plasmodium knowlesi*) and nonhuman primate, rodent, and avian malaria parasite species studied to date (Aurrecoechea et al., 2009).

In this report, we demonstrate that the protein encoded by *PF3D7_1134300* is exported outside the parasitophorous vacuole (PV) to the exofacial surface of the erythrocyte plasma membrane, despite having no canonical export signals, and is also expressed on the surface of merozoites. Based on its distinctive dual surface localization, we designate the protein as *Pf* erythrocyte membrane and merozoite antigen 1 (*Pf*EMMA1) and the corresponding gene as *PfEMMA1*. We show that mouse anti-*Pf*EMMA1 hyperimmune Ig and human affinity-purified anti-*Pf*EMMA1 antibodies restrict parasite growth in vitro. In addition, a recombinant protein–based vaccine derived from the *Plasmodium berghei* (*Pb*) orthologue of *Pf*EMMA1 is immunogenic and can mediate self-cure or prolonged survival in an established uniformly fatal mouse model of severe malaria. Furthermore, high levels of naturally acquired human anti-*Pf*EMMA1 antibodies are associated with significantly lower parasite density in a longitudinal cohort of Tanzanian children. Together, these findings support our hypothesis that antibodies to a novel malaria surface antigen, *Pf*EMMA1, mediate protection against malaria.

## Results

### *Pf*EMMA1 is a highly conserved low-polymorphism parasite protein with a predicted transmembrane domain

**Figure 1.**
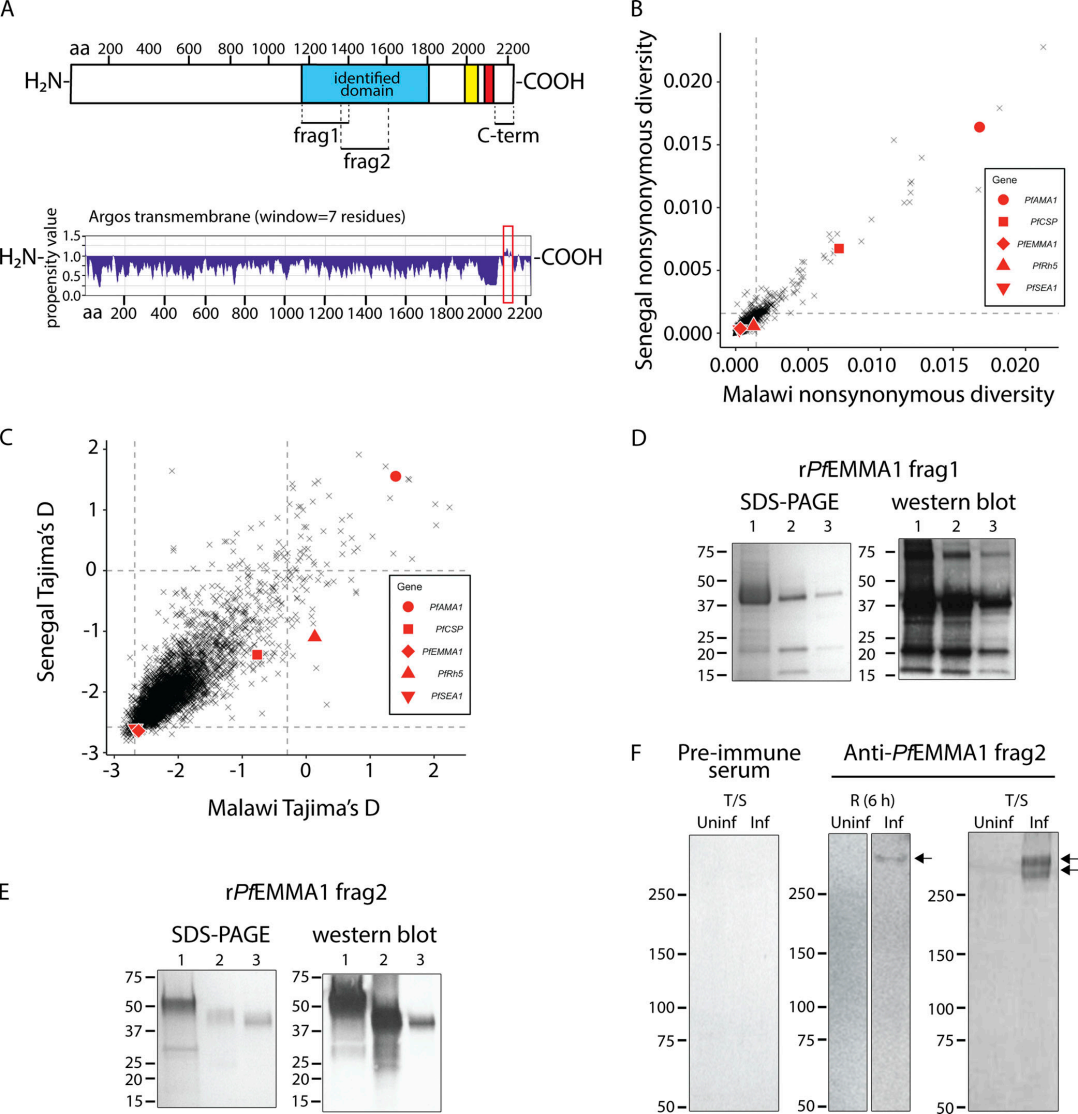
**Characteristics of *Pf*EMMA1.(A)** Predicted protein structure. There is a putative transmembrane helical peptide within the region of aa 2,089–2,135 (red) near the C-terminus as predicted by the Argos transmembrane analytic method (MacVector) and simple tandem repeats shown in yellow. Identified domain refers to the full sequence identified by our phage library screen; fragments 1 (aa 1,164–1,401) and 2 (aa 1,364–1,600) refer to overlapping recombinant polypeptides that were expressed in *E. coli*. C-term refers to the C-terminus region (aa 2,140–2,223). **(B and C)** Conserved genetic sequence. Nonsynonymous diversity of *PfEMMA1* was low in 209 parasites collected from two African countries compared with other known vaccine candidates (B). The Tajima’s *D* statistic is negative, indicating that variants in the gene are primarily at very low frequencies. Dashed lines indicate 2.5% and 97.5% distribution cutoffs (C). **(D and E)** Expression and purification of r*Pf*EMMA1 fragments 1 and 2 from *E. coli* inclusion bodies. Predicted sizes of each fragment are 31 and 32 kD, respectively, although migration of these proteins is slower than expected. Lane 1, urea-solubilized inclusion bodies (r*Pf*EMMA1 fragment 2 has slower migration); lane 2, nickel chelate chromatography of lane 1; lane 3, anion exchange chromatography of lane 2. Equivalent quantities of r*Pf*EMMA1 were loaded for SDS-PAGE and western blots. Amino acid sequences of all bands in lane 3 of SDS-PAGE gels were verified to be fragments of *Pf*EMMA1 by LC-MS/MS. **(F)** Immunoblot of *Pf*3D7-infected erythrocyte lysates. Uninfected (uninf) human RBCs or RBCs infected (inf) with early ring-stage (R) parasites or mixed trophozoites (T)/schizonts (S) were resolved with a 4–15% polyacrylamide gel and probed with murine preimmune sera or anti-*Pf*EMMA1 fragment 2 antisera. Arrows indicate native *Pf*EMMA1 protein. We did not detect any bands using the anti-*Pf*EMMA1 fragment 1 antiserum at concentrations the same as or twofold higher than that of anti-fragment 2 in three independent experiments, presumably because of inadequate sensitivity. Frag, fragment.

In a previously published screen of the *Pf*3D7 blood-stage proteome, we identified a segment of *PF3D7_1134300* (nt 3,490–5,412; aa 1,164–1,804) as uniquely reactive with antibodies in plasma from resistant but not susceptible 2-yr-old Tanzanian children (Raj et al., 2014). Bioinformatics analyses (http://PlasmoDB.org) predict a 263-kD basic phosphoprotein with a single exon (Treeck et al., 2011). 50% of the protein comprises asparagine, glutamic acid, isoleucine, and lysine, and ∼25% of the protein contains low-complexity regions (Wootton, 1994). There are six simple tandem repeats between aa 1,995 and aa 2,060, which is a common feature of exported proteins (Aurrecoechea et al., 2009; Heiber et al., 2013). Functional analyses of the protein sequence did not classify *Pf*EMMA1 in homologous superfamilies, nor did they predict molecular functions or the presence of known domains (InterProScan v5.28-67.0; EMBL-EBI; Jones et al., 2014). *Pf*EMMA1 does not encode a *Plasmodium* export element/host targeting signal (PEXEL/HT) domain, signal peptide, or glycophosphatidylinositol anchor sequence (Petersen et al., 2011; Pierleoni et al., 2008; Sargeant et al., 2006). Unlike many other PEXEL-negative proteins, *Pf*EMMA1 does not contain a conserved N-terminus sequence (Sargeant et al., 2006). However, there is a predicted transmembrane domain near the C-terminus (Fig. 1 A) that corresponds to a hydrophobic region and α-helical structures (Buchan et al., 2013). Gene expression profiling analyses indicate that the gene is expressed throughout the parasite erythrocytic stages, with a higher detection of the transcript at schizont stages (Bozdech et al., 2003; Le Roch et al., 2003; Otto et al., 2010; Toenhake et al., 2018). Polysome and ribosome profiling analyses suggest that the protein is actively translated during both the ring and schizont stages (Bunnik et al., 2013; Caro et al., 2014), with a higher expression at the ring stage of the erythrocytic life cycle.

**Figure S1.**
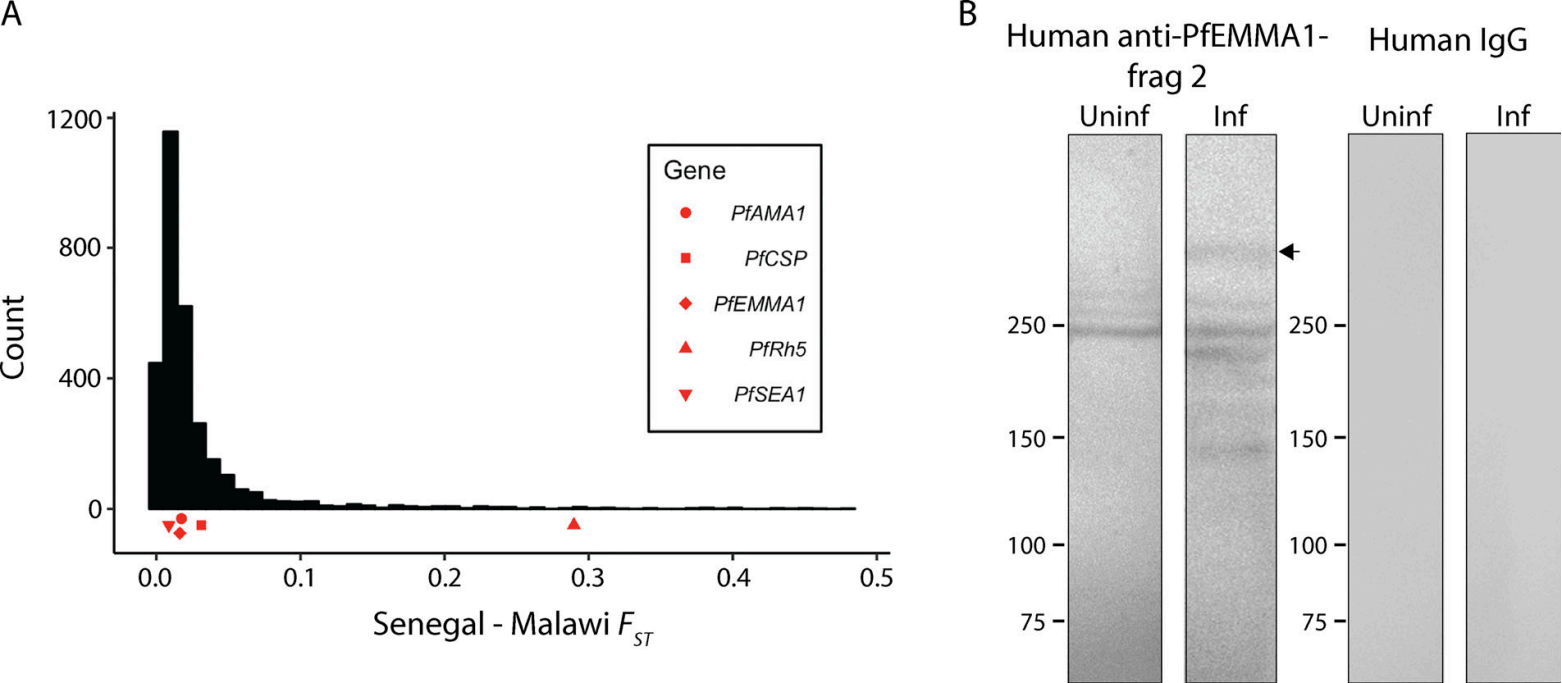
***Pf*EMMA1 differentiation at the population level and immunoblot of *Pf*3D7-infected erythrocyte lysates probed with human antibodies. (A)** The Weir and Cockerham’s estimate of *F_ST_* between Senegal (West Africa) and Malawi (southeastern Africa) for *Pf*EMMA1 is similarly low compared with certain other vaccine candidates, indicating a low degree of genetic variance between parasite populations. **(B)** Immunoblot of *Pf*3D7-infected erythrocyte lysate probed with human antibodies. Uninfected (uninf) human RBCs and RBCs infected (inf) with predominantly ring-stage parasites were resolved with a 4–15% polyacrylamide gel and probed with affinity-purified human anti-*Pf*EMMA1 polyclonal Igs or malaria-naive human IgG (each 1 mg/ml). The arrow indicates full-length native *Pf*EMMA1 protein.

To examine the genetic diversity of the gene in freshly isolated parasites, we analyzed the *Pf* gene sequences of single-clone lineages from samples collected in Senegal and Malawi from the MalariaGen Pf3k project (Manske et al., 2012). Single nucleotide polymorphisms (SNPs) of *PfEMMA1* were compared with those of *PfAMA1*, *PfCS*P, *PfRH5*,**and *PfSEA-1* after applying stringent quality-control filters. *PfEMMA1* shows average levels of pairwise nonsynonymous nucleotide diversity (π_NS_: 3.6 × 10^−4^, 55th percentile in Senegal; 3.2 × 10^−4^, 52nd percentile in Malawi), which contrasts with the high nucleotide diversity present in *PfCSP* and *PfAMA1* (99th percentiles in Senegal and Malawi for each gene; Fig. 1 B). In addition, the value of Tajima’s *D*, a diversity-based statistic that can detect selection and/or demographic changes, is very low for *PfEMMA1* in both populations (−2.64, second percentile in Senegal; −2.62, sixth percentile in Malawi; Fig. 1 C). This indicates an excess of low-frequency variants, a pattern suggesting that *PfEMMA1* may be undergoing purifying selection. This selection appears uniform across geographic regions, as population differentiation between West and East Africa (as measured by *F_ST_*) is not elevated (62nd percentile; Fig. S1). Using an expanded set of 1,315 globally distributed, single-clone infections from the Pf3k project, we confirmed that low polymorphism is maintained outside Senegal and Malawi, as only a single SNP has a global minor allele frequency (MAF) >5% (nt 294, MAF = 0.118). Within our identified *PfEMMA1* segment (nt 3,490–5,412), we did not detect any SNPs at a frequency >5% and only five with a combined MAF >1% (Caro et al., 2014). These data indicate that *PfEMMA1* contains little amino acid variation at both local and global scales.

### *Pf*EMMA1 is present in Maurer’s clefts (MC) and on RBC surface membranes

To validate the protein expression of *PfEMMA1,* we generated antibodies to two overlapping fragments of *Pf*EMMA1 that were contained within the segment originally identified in our whole-proteome differential screen (Raj et al., 2014). Specifically, we immunized BALB/cJ mice with two codon-optimized, recombinantly expressed, and purified polypeptides (fragments 1 and 2; 38-aa overlap; Fig. 1, D and E). We confirmed that *PfEMMA1* encodes a native parasite protein by probing lysates of *Pf*3D7-infected RBCs with murine anti-*Pf*EMMA1 fragment 2 antiserum. We detected a single protein band in early ring-stage parasites and a double protein band in mixed trophozoite-/schizont-stage parasites using western blot analyses (Fig. 1 F). The single (MW, 308 kD) and double (MW, 289 and 304 kD) band sizes approximate the calculated MW (263 kD) for unmodified full-length *Pf*EMMA1. We propose an explanation for the double band below. We then demonstrate in an immunoblot that affinity-purified anti-*Pf*EMMA1 polyclonal Ig from plasma of Kenyan adults detected a single band (MW, 305 kD) consistent with full-length *Pf*EMMA1 in predominantly ring-stage *Pf*3D7-infected RBCs (Fig. S1 B).

**Figure S2.**
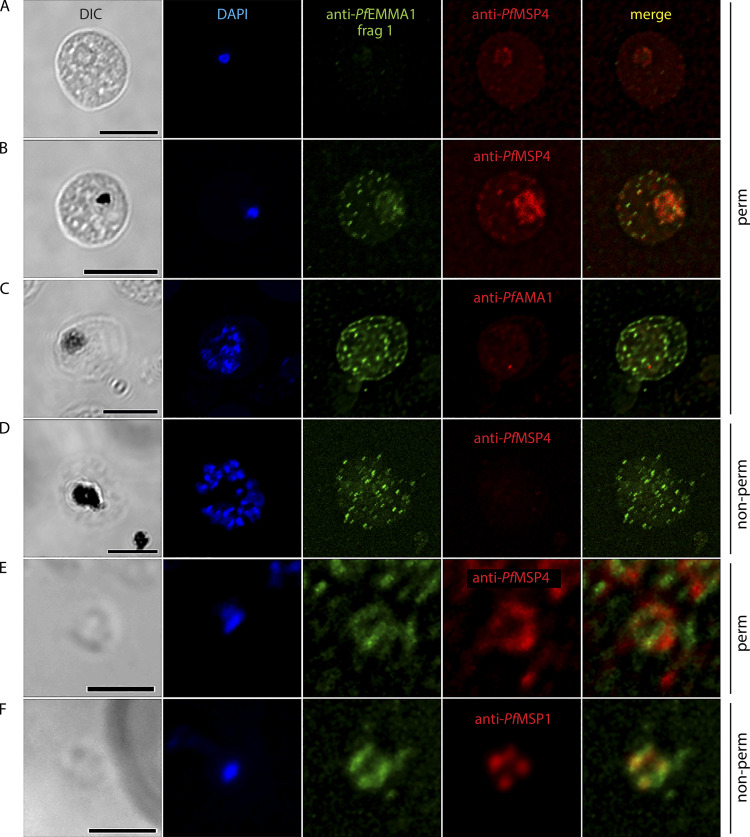
**Structural and temporal localization of *Pf*EMMA1 by immunofluorescence confocal microscopy. (A–C)** Permeabilized *Pf*3D7-infected RBCs were probed with mouse anti-*Pf*EMMA1 fragment 1 (green) and rabbit anti-*Pf* merozoite surface protein 4 (MSP4; red) or *Pf* apical membrane antigen 1 (AMA1; red) antibodies and counterstained with DAPI to label parasite nuclei. *Pf*EMMA1 is detected after the ring stage, when it is expressed in a stippled pattern outside the parasite membrane and at the RBC periphery. **(D)** Exofacial surface labeling of *Pf*EMMA1 is shown in a live, nonpermeabilized *Pf*3D7-infected RBC probed with mouse anti-*Pf*EMMA1 fragment 1 (green) and rabbit anti-*Pf* merozoite surface protein 4 (MSP4; red). **(E** **and F)** *Pf*3D7 merozoites that were either permeabilized (E) or live, nonpermeabilized (F) were probed with antibodies against *Pf*EMMA1 fragment 1. Counterstains included DAPI and antibodies to *Pf*MSP1 or *Pf*MSP4. DIC, differential interference contrast microscopy. Scale bar, 5 µm (A–D) or 2 µm (E and F).

**Figure 2.**
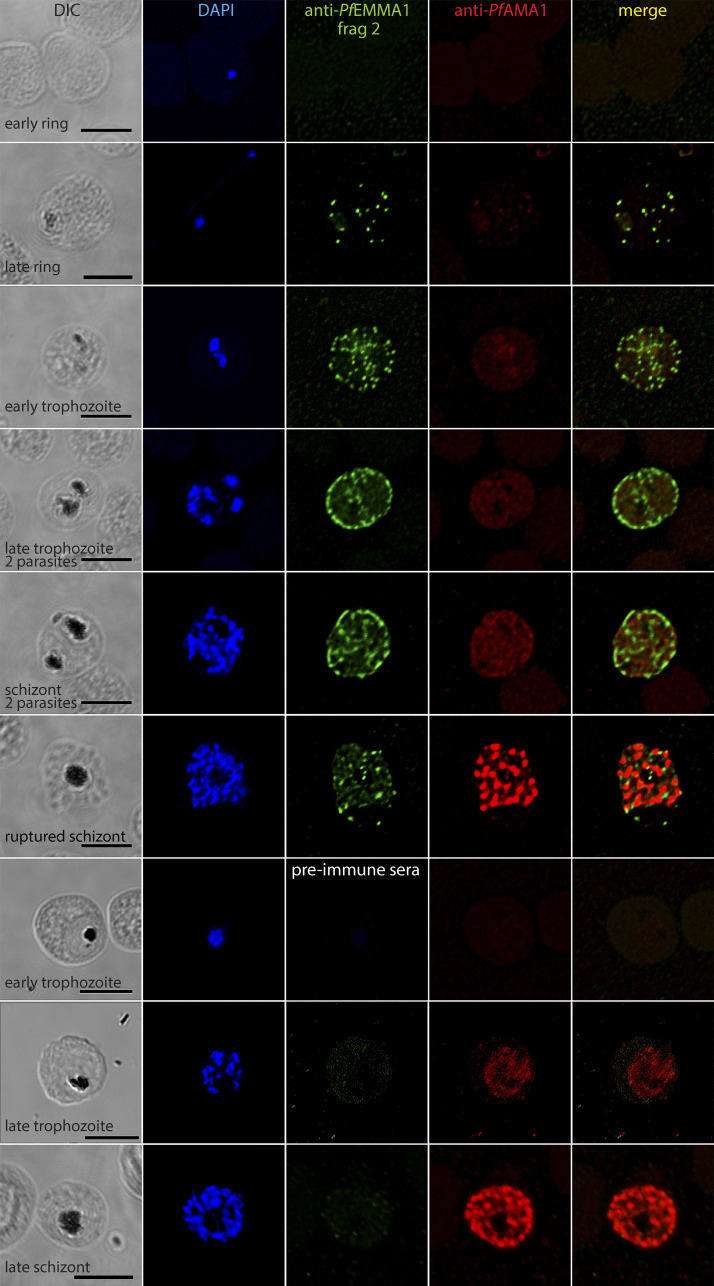
**Structural and temporal localization of *Pf*EMMA1 by immunofluorescence confocal microscopy using anti-*Pf*EMMA1 fragment 2 antisera.** Representative permeabilized *Pf*3D7-infected RBCs were probed with mouse anti-*Pf*EMMA1 fragment 2 (green) or preimmune sera and rabbit anti-*Pf*AMA1 (red) and counterstained with DAPI to label parasite nuclei. *Pf*EMMA1 is detected in all infected RBCs after the ring stage, when it is expressed in a stippled pattern outside the parasite, and then it aggregates at the RBC periphery but does not colocalize with *Pf*AMA1, a merozoite marker. No proteins were detected with preimmune sera. Scale bar, 5 µm. DIC, differential interference contrast microscopy.

**Figure 3.**
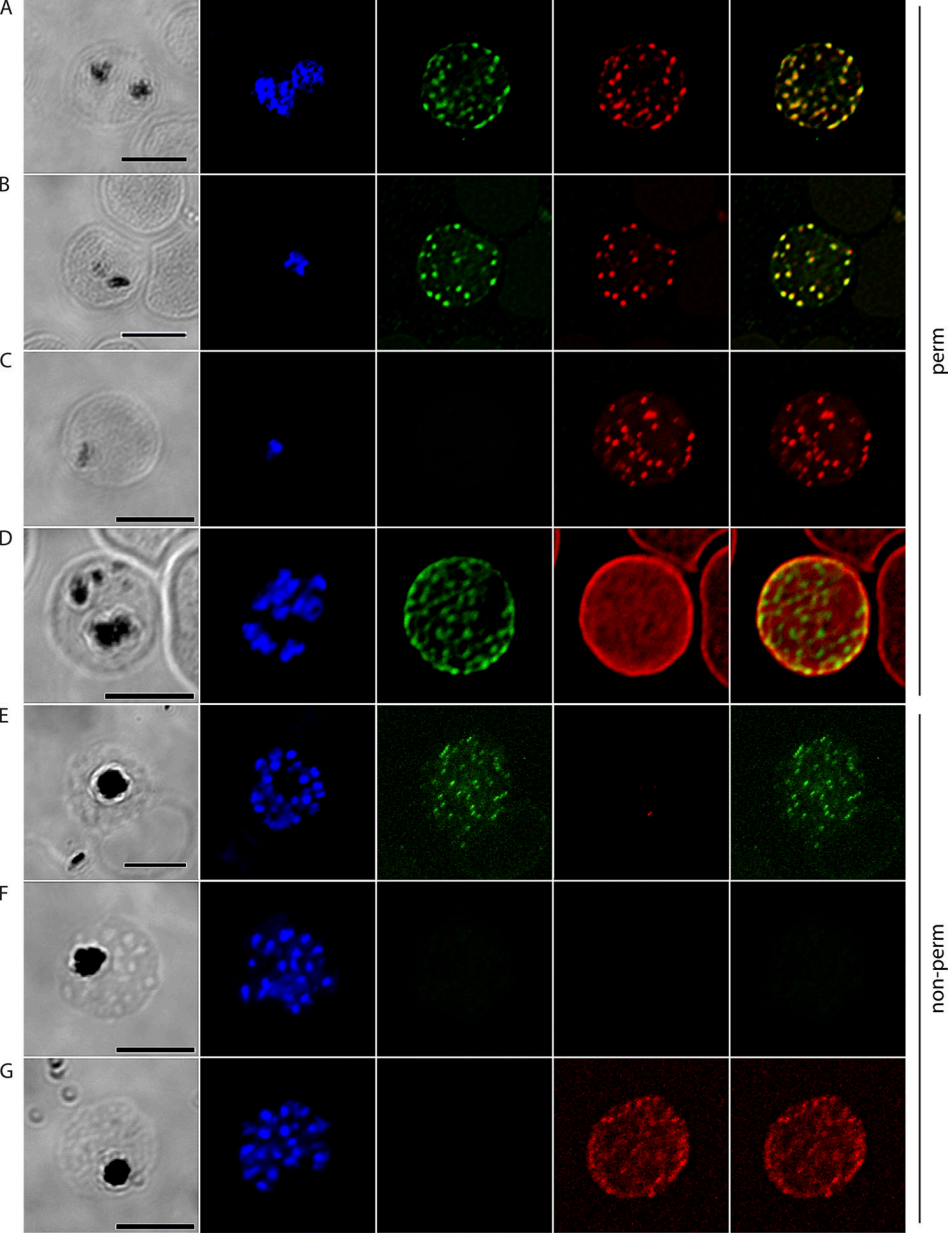
**Erythrocyte surface localization of *Pf*EMMA1 by immunofluorescence confocal microscopy. (A–E)** Representative *Pf*3D7-infected RBCs were probed with mouse anti-*Pf*EMMA1 fragment 2 (green) and rabbit antibodies against various parasite or host proteins (red) and counterstained with DAPI to label parasite nuclei. *Pf*EMMA1 colocalizes with REX1 (A) and SBP1 (B), structural proteins of MC, in permeabilized iRBCs. A concurrent control experiment with mouse preimmune sera is shown (C). *Pf*EMMA1 colocalizes with GPA, a host cell surface glycoprotein, in permeabilized iRBCs (D). In live, nonpermeabilized iRBCs, anti-*Pf*EMMA1 fragment 2 antibodies specifically labeled proteins on the exofacial surface of 8.5% of ~2,000 RBCs (E); rabbit anti-*Pf*MSP4 antibodies did not penetrate the RBC surface membrane. **(F and G)** Concurrent control experiments with mouse preimmune sera are shown counterstained with anti-*Pf*MSP4 antibodies, which do not penetrate the RBC surface membrane (F), and anti-GPC, which labels exofacial surface glycoproteins (G). Scale bar, 5 µm. DIC, differential interference contrast microscopy; perm, permeabilized; nonperm, nonpermeabilized; *Pf*MSP4, merozoite surface protein 4; *Pf*REX1, ring exported protein 1; *Pf*SBP1, skeleton binding protein 1.

To determine the cellular localization of *Pf*EMMA1, we performed immunofluorescence (IF) confocal microscopy on permeabilized iRBCs using antiserum against *Pf*EMMA1 fragment 1 (Fig. S2, A–C) and fragment 2 (Fig. 2). We detected punctate IF structures outside the parasite’s plasma membrane. Using dual IF labeling against *Pf*EMMA1 and ring-exported protein-1 (REX1; Fig. 3 A) or skeleton-binding protein 1 (SBP1; Fig. 3 B), both structural components of MC, we showed colocalization of these proteins in the punctate structures, whereas no proteins were labeled with preimmune sera (Fig. 3 C). The export of *Pf*EMMA1 outside the parasite plasma membrane, despite the lack of canonical export signals, categorizes this protein as a PEXEL-negative exported protein (PNEP). The identification of a predicted transmembrane domain suggests that translocation of this protein is most likely the mode of delivery into the host cell (Heiber et al., 2013).

In addition, we demonstrated that *Pf*EMMA1 is concentrated at the periphery of permeabilized trophozoite- and schizont-stage iRBCs by IF assays using anti-*Pf*EMMA1 fragment 1 (Fig. S2 C) and fragment 2 (Fig. 3 D) antibodies. We also showed that *Pf*EMMA1 is closely associated with glycophorin A (GPA) detected using an anti-GPA antibody that specifically recognizes an intracellular GPA epitope (Fig. 3 D). To determine whether *Pf*EMMA1 is exposed on the exofacial surface of the RBC plasma membrane, we probed live, nonpermeabilized, schizont-infected RBCs with anti-*Pf*EMMA1 fragment 1 (Fig. S2 D) and fragment 2 (Fig. 3 E) antibodies. We observed a diffuse stippled staining pattern with anti-*Pf*EMMA1 antibodies that was not detected using preimmune sera (Fig. 3, F and G). Counterstains with antibodies to *Pf*MSP4 (Fig. 3 F) and glycophorin C (GPC; Fig. 3 G) confirmed that the RBC plasma membrane was impermeable, indicating that native *Pf*EMMA1 is exposed on the exofacial surface of infected RBCs.

**Figure 4.**
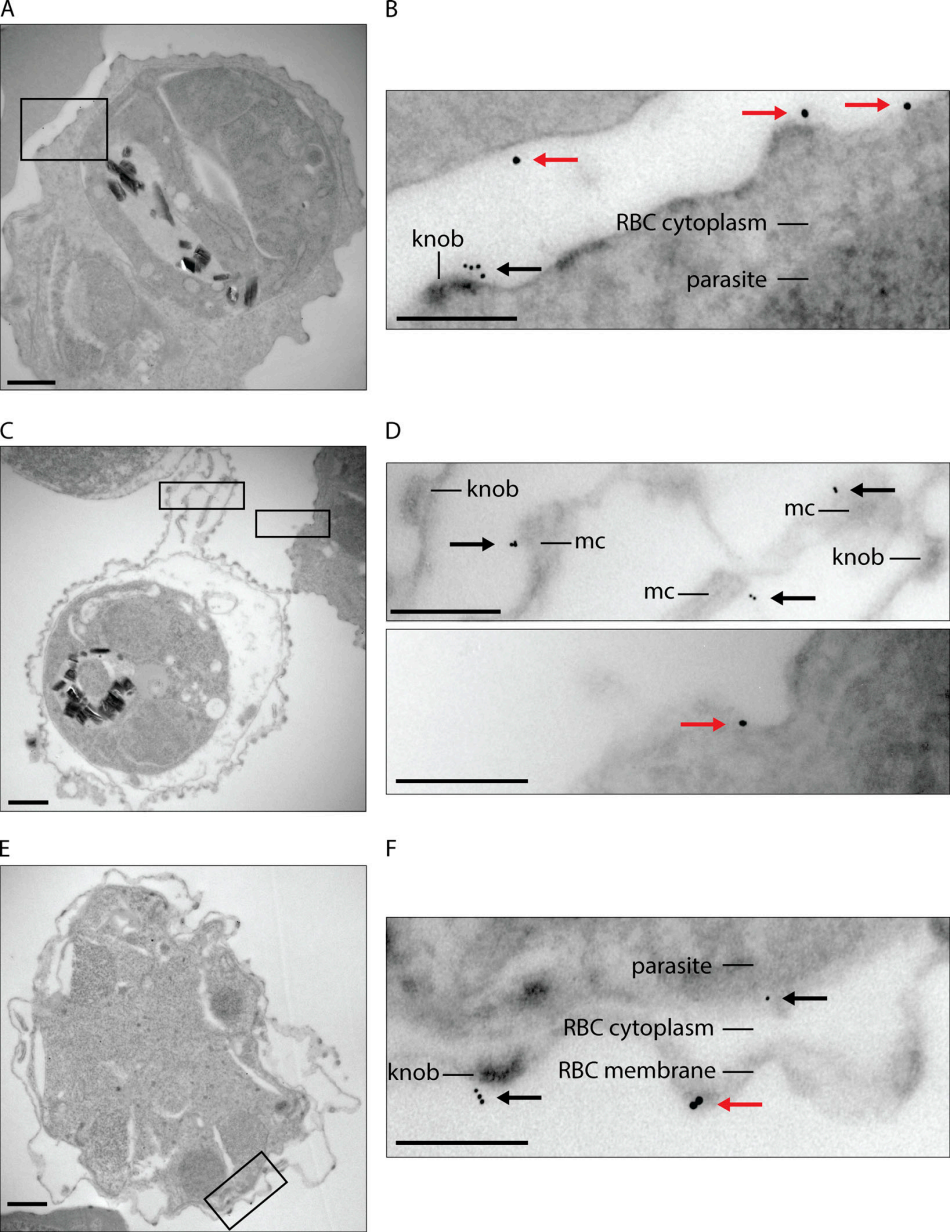
**Localization of *Pf*EMMA1 with immunogold electron microscopy.** Late-stage infected RBCs were probed with mouse anti-*Pf*EMMA1 fragment 2 antibodies (6-nm gold particles; black arrows) and rabbit anti-GPC (10-nm gold particles; red arrows). **(A and B)** In a representative live, non-permeabilized iRBC, *Pf*EMMA1 localized to the exofacial surface of the RBC membrane in close proximity to a knob with a characteristic electron-dense layer under the RBC surface membrane. Approximately 7% of infected RBCs had evidence of exofacial surface labeling. (**C–F**) In permeabilized iRBCs, *Pf*EMMA1 localized to MCs, the parasite surface, and an electron-dense knob on the iRBC surface membrane. Scale bars, 0.5 µm (A, C, and E) or 200 nm (B, D, and F).

To confirm the surface localization of *Pf*EMMA1, we performed immuno-transmission electron microscopy studies. We used anti-*Pf*EMMA1 fragment 2 antibodies because they demonstrated superior sensitivity in the immunoblot and IF assays (Fig. 1 F; Fig. 3, D and E; and Fig. S2, C and D). We detected *Pf*EMMA1 labeled with clusters of immunogold-conjugated anti-*Pf*EMMA1 antibodies in various locations: (a) on the exofacial surface of live, nonpermeabilized iRBCs in close proximity to a knob (Watermeyer et al., 2016), with its characteristic electron-dense layer underlying the RBC membrane (Fig. 4, A and B), and (b) on the surface of MCs within an iRBC (Fig. 4, C and D), on an electron-dense knob and on the surface of an intact parasite (Fig. 4, E and F) within permeabilized parasites treated with equinatoxin II (EqtII; Jackson et al., 2007). In simultaneous control experiments, preimmune sera did not label any proteins in live, nonpermeabilized, or permeabilized iRBCs.

### *Pf*EMMA1 is expressed on the merozoite surface

**Figure 5.**
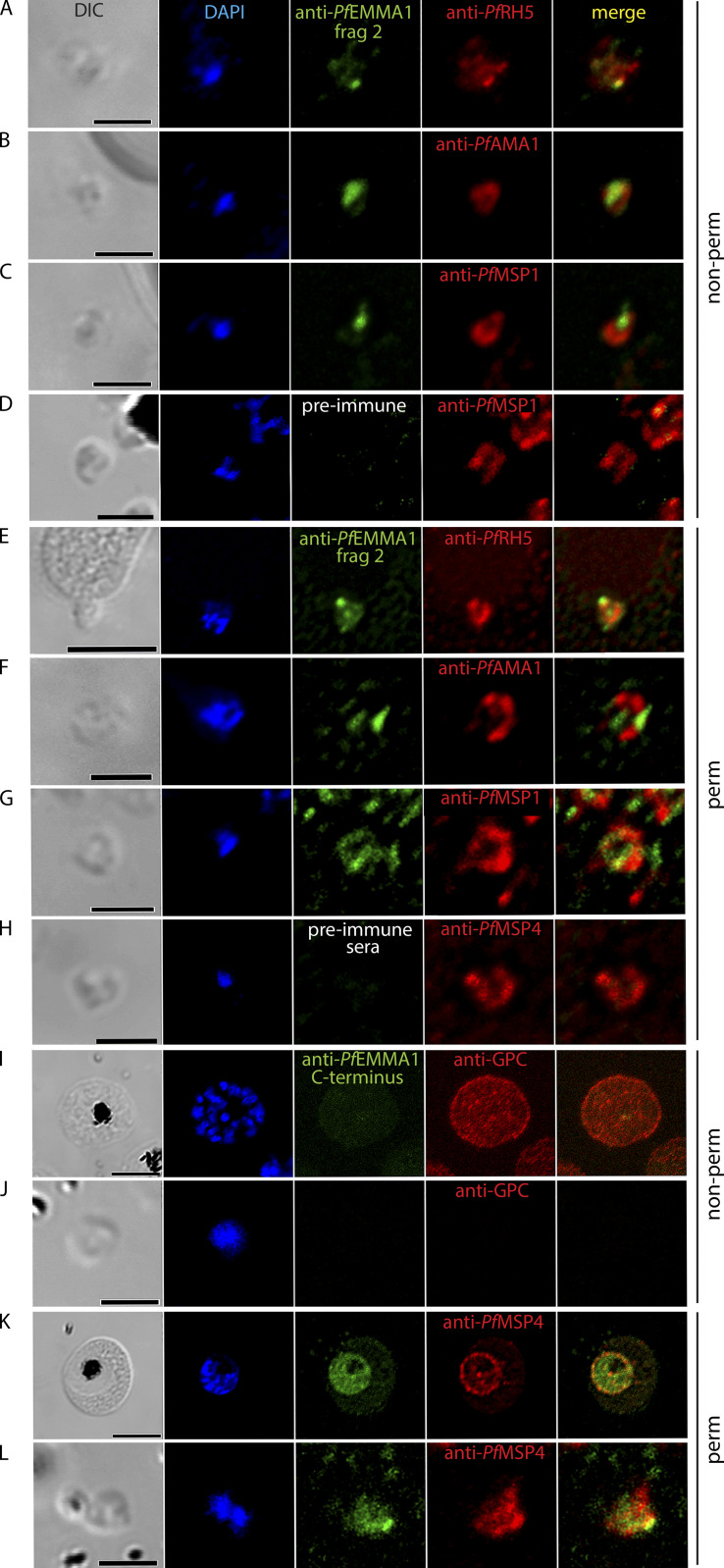
**Subcellular distribution of *Pf*EMMA1 in merozoites and infected RBCs.(A–H)** Merozoites (*Pf*3D7) that were live, non-permeabilized (A–D), or permeabilized (E–H) were probed with antibodies against *Pf*EMMA1 fragment 2 (A–C and E–G), and labeling of *Pf*EMMA1 is shown. No proteins were detected with preimmune serum (D and H). A merozoite is in contact with an uninfected RBC at its apex where *Pf*EMMA1 is highly expressed (E). **(I–L)** A live, non-permeabilized *Pf*3D7 schizont (I) and merozoite (J) were compared with a permeabilized schizont (K) and merozoite (L); cells were probed with anti-*Pf*EMMA1 C-terminus antibodies (I–L). Staining of *Pf*EMMA1 was observed only in permeabilized iRBCs (K) and merozoites (L). Counterstains included DAPI, *Pf*AMA1, *Pf*MSP1 and ˗4, *Pf*RH5, and anti-GPC. DIC, differential interference contrast microscopy. Scale bars, 2 µm (A–D, F–H, J, and L) or 5 µm (E, I, and K).

To identify *Pf*EMMA1 on parasites, we applied an IF confocal microscopy assay using antibodies targeting *Pf*EMMA1 fragment 2. We identified the protein on the exofacial surface of non-permeabilized merozoites in close association with *Pf*RH5 (rhoptry protein), but remote from *Pf*AMA1 (microneme protein) and *Pf*MSP1 (surface protein; Pearson’s correlation coefficient <0.5 for each; Fig. 5, A–C). We were unable to detect any proteins using preimmune sera (Fig. 5 D). *Pf*EMMA1 was visualized in permeabilized merozoites (Fig. 5, E–G) that was not detected with preimmune sera (Fig. 5 H). In certain fields (Fig. 5, C and E), *Pf*EMMA1 appears to be localized to the apex of merozoites and is oriented in close proximity to the site of contact with an uninfected RBC (Fig. 5 E). Antibodies to *Pf*EMMA1 fragment 1 showed similar staining patterns (Fig. S2, E and F).

### Truncated *Pf*EMMA1 is exported but full-length protein is confined to the parasite

**Figure S3.**
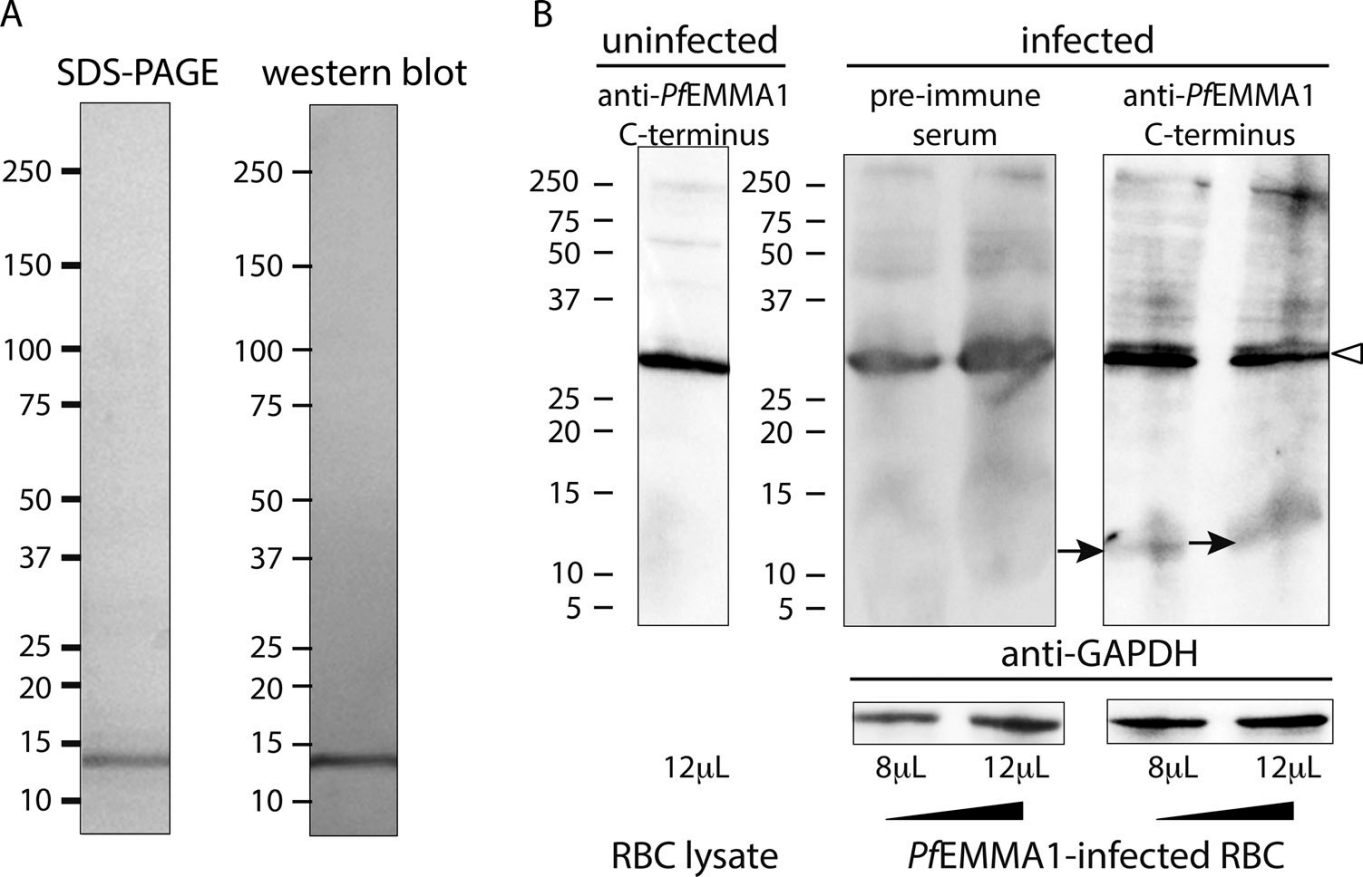
**Purification and expression of *Pf*EMMA1 C-terminus.(A)** Purification of r*Pf*EMMA1 C-terminus from *E. coli* inclusion bodies was achieved with sequential fast protein liquid chromatography using nickel chelate affinity chromatography and anion exchange chromatography. The protein was resolved by SDS-PAGE, and the amino acid sequence was verified by LC-MS/MS. Western blot detected a single protein band of the expected size (S⋅tag-r*Pf*EMMA1 C-terminus-10x His fusion protein: observed size, 13.8 kD vs. calculated size, 13.2 kD). **(B)** Denatured lysates (8 or 12 µl) of RBCs infected with synchronized early trophozoite stage *Pf*3D7 parasites (8% parasitemia) or uninfected human RBCs were resolved in an immunoblot with a 16.5% polyacrylamide gel and probed with murine preimmune serum or anti-*Pf*EMMA1 C-terminus antiserum. *Pf*GAPDH was used as a loading control. Solid arrows indicate the C-terminus fragment of native *Pf*EMMA1 protein (11.5 kD). Arrowhead indicates nonspecific labeling of an endogenous RBC protein.

To further investigate the dynamics of *Pf*EMMA1 export, we expressed and purified a codon-optimized C-terminus recombinant peptide (Fig. S3 A) and generated murine polyclonal anti-*Pf*EMMA1 C-terminus antibodies. In contrast to the exofacial surface localization of *Pf*EMMA1 detected by anti-*Pf*EMMA1 fragment 1 and 2 antibodies that target epitopes on the N-terminus side of the predicted transmembrane domain, polyclonal antibodies targeting epitopes in the C-terminus region immediately downstream of the predicted transmembrane domain failed to detect *Pf*EMMA1 on the external surface of non-permeabilized iRBCs (Fig. 5 I) or merozoites (Fig. 5 J) in the same assay. On the other hand, the anti-*Pf*EMMA1 C-terminus antibodies did label *Pf*EMMA1 within the parasite plasma membrane in permeabilized fixed iRBCs (Fig. 5 K), as well as within permeabilized fixed merozoites (Figs. 5 L). These observations suggest that full-length *Pf*EMMA1, which contains the C-terminus region, is not exported to the surface but instead is confined within the intraerythrocytic parasite and merozoite surface membranes, whereas postulated cleavage of the C-terminus at or near the transmembrane domain leads to export of a truncated *Pf*EMMA1 to the RBC and merozoite surface membranes. In support of this hypothesis, native *Pf*EMMA1 was identified by western blot as a single protein band in early ring-stage parasites and as two protein bands in trophozoite-/schizont-stage parasites (Fig. 1 F). The double bands differ in size by 13 kD, which approximates the calculated size of the region from the putative transmembrane domain to the distal C-terminal end (10.1 kD). Furthermore, anti-*Pf*EMMA1 C-terminus antibodies labeled a large band (∼260 kD on 16.5% polyacrylamide Tris-Tricine gel) as well as a 11.5-kD protein fragment in lysates of synchronized early trophozoite-stage iRBCs on the same gel (Fig. S3 B). The observed size of the small fragment closely approximates the calculated size of the C-terminus region. Taken together, these findings are consistent with a proposed mechanism by which full-length *Pf*EMMA1 is retained within the parasite throughout the erythrocytic cycle, whereas removal of the *Pf*EMMA1 C-terminus region at a slightly later stage of intraerythrocytic development mediates export of the truncated protein to the RBC surface.

### *Pf*EMMA1 is not essential for in vitro parasite survival, but is the target of growth-inhibiting anti-*Pf*EMMA1 antibodies

**Figure 6.**
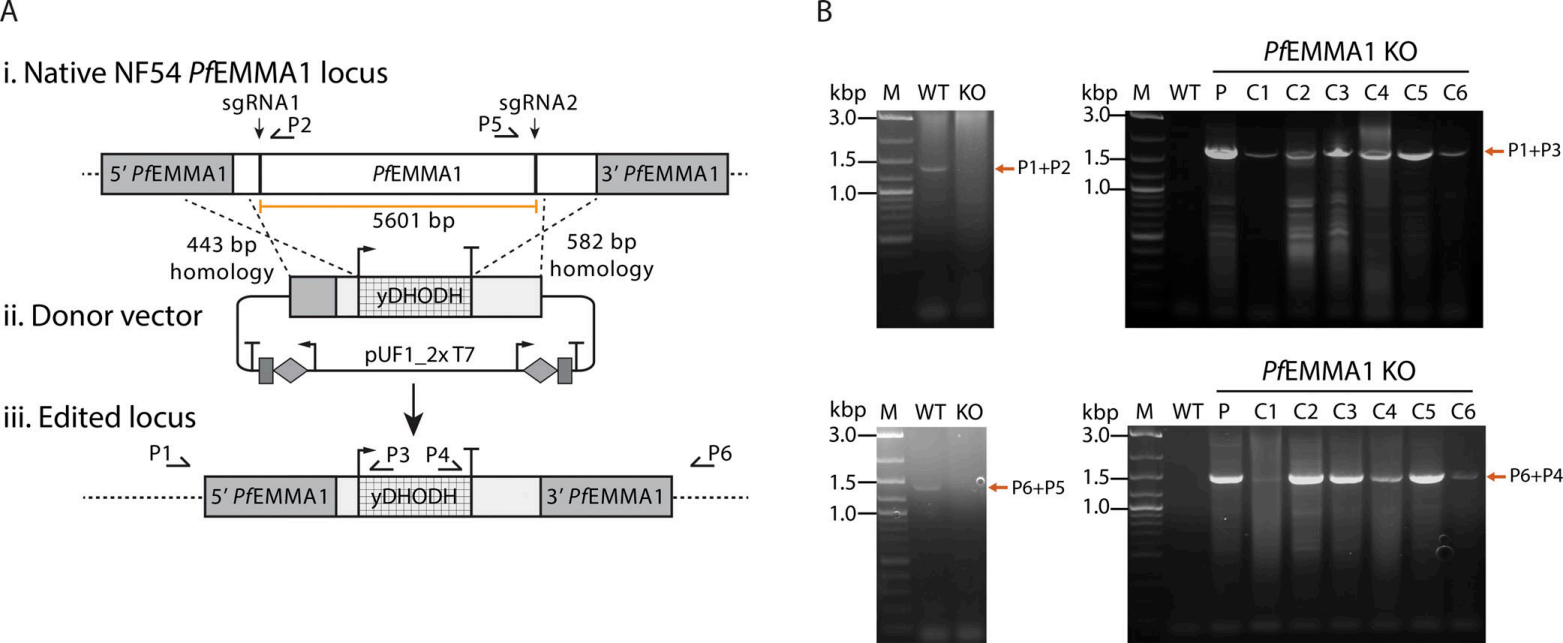
**Construction and characterization of NF54 *Pf*EMMA1-KO parasites.(A)** Schematic of the pCRISPR-Cas9–mediated deletion of the native NF54 *PfEMMA1* locus and replacement with the *yDHODH* gene from the pUF1-2xT7 vector containing two T7 cassettes for expression of two guide RNAs. **(B)** Diagnostic PCR analysis of WT and KO clones. Loss of the native locus was assessed using primers P1 and P2 that amplify a 1,404-bp fragment at the 5′-end and P6 and P5 that amplify a 1,450-bp fragment at the 3′-end. Integration of the *PfEMMA1_*KO donor vector was assessed using primers P1 and P3 that amplify a 1,744-bp fragment at the 5′-end and P6 and P4 that amplify a 1,502-bp fragment at the 3′-end. C1˗6, clonal *PfEMMA1* KO parasites; M, 2-log DNA ladder; Parental, nonclonal *PfEMMA1* KO parasites; P, parental strain; WT, NF54^attB^ gDNA.

To test whether *Pf*EMMA1 is essential for erythrocytic-stage parasite growth, we used CRISPR/Cas9 genome engineering to delete the gene in a *Pf*NF54 strain background (Fig. 6 A and Table S1). We recovered viable, clonal parasites that were verified to be deleted in *PfEMMA1* (Fig. 6 B). These data are consistent with a recently reported *piggybac*-transposon mutagenesis screen that similarly classified *Pf*EMMA1 as dispensable to parasite survival during development in RBCs in vitro (Zhang et al., 2018).

**Figure 7.**
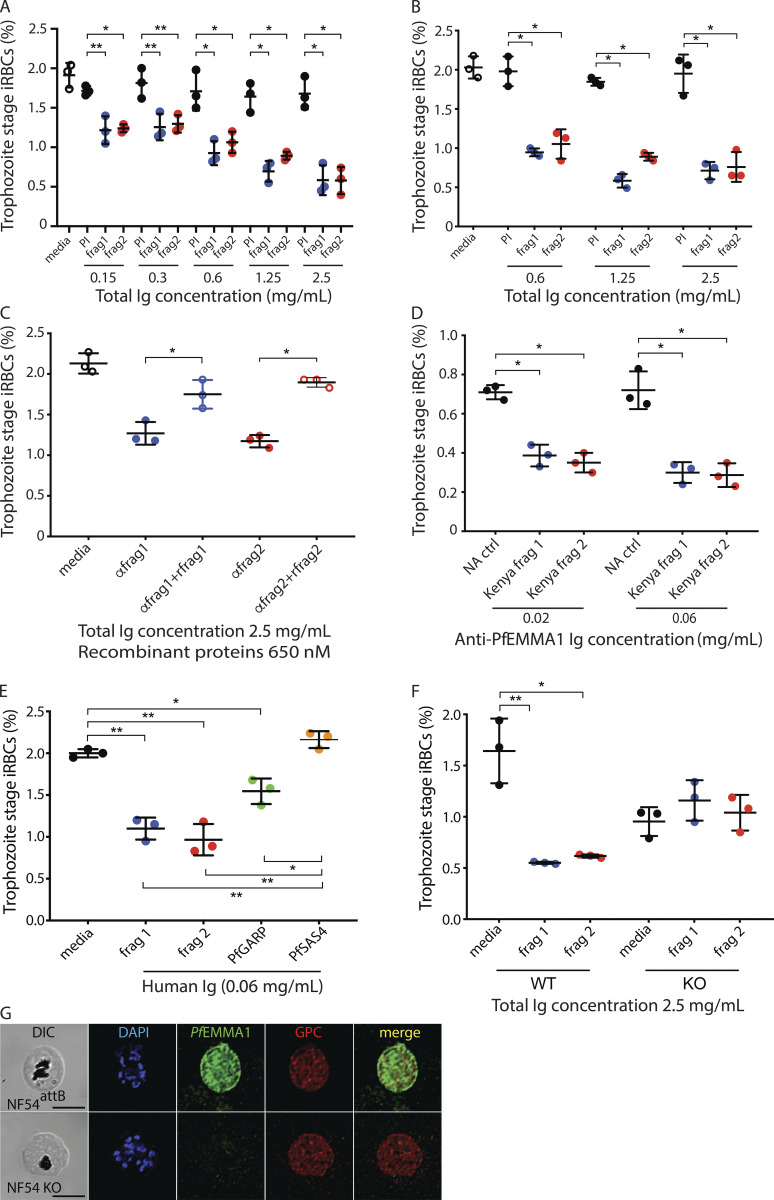
**Restriction of malaria parasite growth by antibodies to *Pf*EMMA1 and *Pf*EMMA1 KO phenotype.(A and B)** Purified polyclonal mouse anti-r*Pf*EMMA1 Igs inhibited the growth and invasion of *Pf* homologous (A, 3D7) and heterologous (B, Dd2) strains ≤68% in a dose-dependent manner in GIA assays. Experiments were performed in triplicate and are representative of three independent assays. *, P < 0.003; **, P < 0.03. PI, preimmune sera. **(C)** The neutralizing effect of *Pf*EMMA1 Igs on *Pf*3D7 parasites was reversed by preincubating antibodies (anti-fragment 1 [αfrag1] and αfrag2, 2.5 mg/ml each) with recombinant proteins (recombinant fragment 1 [rfrag1] and rfrag2, respectively; 650 nM each). Experiments were performed in triplicate and are representative of three independent experiments. *, P < 0.003. **(D)** Specific affinity-purified anti-*Pf*EMMA1 fragment 1 and 2 polyclonal Igs from the plasma of immune Kenyan adults inhibited 3D7 in GIAs significantly more than serum from North American (NA) controls. Experiments were performed in triplicate and are representative of three independent assays. Distribution of data is shown by mean and SD. *, P < 0.003. **(E)** Affinity-purified Kenyan adult anti-*Pf*EMMA1 fragment 1 and 2 polyclonal Igs inhibited 3D7 in GIAs significantly more than medium and anti-*Pf*SAS4 Ig (negative control) and similarly to anti-*Pf*GARP Ig (positive control). All Igs (0.06 mg/ml) were derived from the same individual’s plasma. Experiments were performed in triplicate and are representative of two independent assays. Distribution of data is shown by mean and SD. *, P < 0.01; **, P < 0.001. **(F)** Purified murine polyclonal Igs (2.5 mg/ml) raised against *Pf*EMMA1 fragments 1 and 2 inhibited parasite invasion of WT parasites (*, P < 0.01; **, P < 0.008) but not KO parasites, which had a slower replication rate. These results are consistent with the –1.4 mutant fitness score reported in the *piggybac*-transposon mutagenesis screen (Zhang et al., 2018). Experiments were performed in triplicate and are representative of three independent experiments. Differences in parasite density for all experiments (A–F) were analyzed using Student’s *t* test. **(G)** Immunofluorescence confocal microscopy. Permeabilized RBCs infected with NF54^attB^ or KO parasites at schizont stage were probed with mouse anti-*Pf*EMMA1 fragment 2 and rabbit anti-GPC antibodies and counterstained with DAPI to label parasite nuclei. The KO phenotype is representative of iRBCs at 6% parasitemia observed in 25 high-power fields. Scale bar, 5 µm. DIC, differential interference contrast microscopy; frag, fragment; WBC, white blood cell.

**Figure S4.**
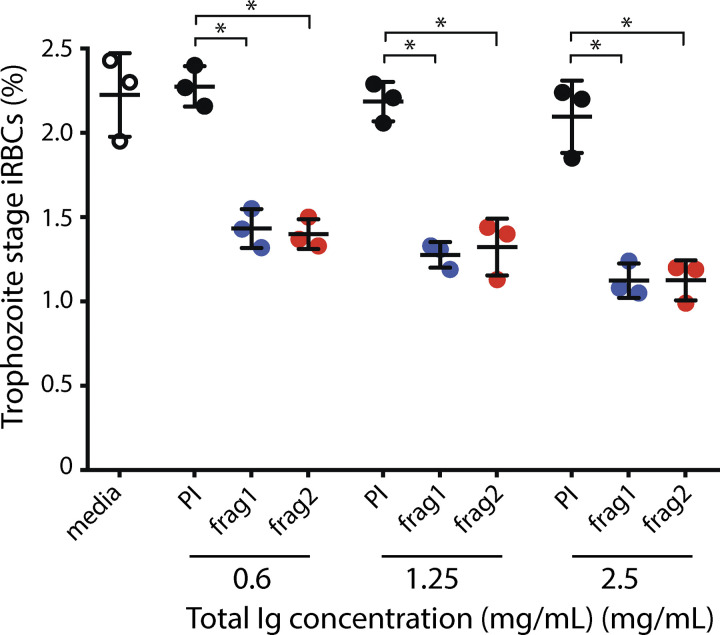
**Inhibition of *Pf*W2 strain by antibodies to *Pf*EMMA1.** Purified Igs to *Pf*EMMA1 recombinant proteins significantly inhibited parasite growth/invasion for *Pf*W2 strain in a dose-dependent manner (*, P < 0.003). PI, preimmune serum; frag 1, anti-*Pf*EMMA1 fragment 1 antibodies; frag 2, anti-*Pf*EMMA1 fragment 2 antibodies.

While *Pf*EMMA1 function appears to be dispensable for parasite growth in vitro, we sought to test whether it could mediate antibody-dependent inhibition of parasite growth. We performed growth inhibition activity (GIA) assays using total polyclonal Igs purified from mice immunized with recombinant (r)*Pf*EMMA1 fragments (strain 3D7). Two heterologous *Pf* strains (3D7 and Dd2) and a strain related to Dd2 (W2) were cultivated with RBC-predepleted anti-r*Pf*EMMA1 or preimmune antibodies. *Pf*3D7 parasite growth was inhibited by ≤68% compared with control antibodies in a dose-dependent manner (P < 0.003; Fig. 7 A). GIA assays with *Pf*Dd2 (Fig. 7 B) and *Pf*W2 (Fig. S4) strains showed inhibition ≤67% and ≤47%, respectively. The half maximal effective concentration (EC_50_) values for total Ig enriched for anti-*Pf*EMMA1 fragment 1 and 2 antibodies were 0.52 and 0.71 mg/ml, respectively. We confirmed that the inhibitory effect of Ig was attributable solely to anti-*Pf*EMMA1–specific antibodies by demonstrating a significant reversal of GIA due to competitive neutralization when polyclonal Ig was preincubated with cognate r*Pf*EMMA1 fragments (P < 0.003; Fig. 7 C). Similarly, affinity-purified anti-*Pf*EMMA1 Ig from an immune Kenyan adult resulted in ≤60% growth inhibition in comparison with Ig from North American controls (Fig. 7 D). In separate experiments, Kenyan affinity-purified anti-*Pf*EMMA1 Ig significantly inhibited parasite growth relative to media alone or affinity-purified human anti-*Pf*SAS4 Ig, which has no surface expression; Kenyan anti-*Pf*GARP Ig purified from the same plasma was used as a positive control (Fig. 7 E; Raj et al., 2020).

To test the absolute requirement for *Pf*EMMA1 to mediate these growth inhibitory effects, we performed GIA assays on the *PfEMMA1* KO line. While total Ig from murine antisera inhibited growth of the parental *Pf*NF54 strain, no inhibition of *PfEMMA1*-KO parasite growth was observed (Fig. 7 F). In contrast to the control parental line, we did not detect *Pf*EMMA1 immunofluorescence signal for *PfEMMA1*-KO parasites, confirming the lack of immunoreactivity of these parasites with anti-*Pf*EMMA1 antibodies (Fig. 7 G). Altogether, these data confirm that while *Pf*EMMA1 is dispensable to blood-stage parasite growth, expression of this antigen and its specific recognition by anti-*Pf*EMMA1 antibodies mediate significant inhibition of blood-stage parasite growth in vitro.

### Immunization of mice with recombinant *Pb*EMMA1 (r*Pb*EMMA1) can mediate self-cure or prolonged survival after challenge with uniformly lethal *Pb*ANKA

**Figure S5.**
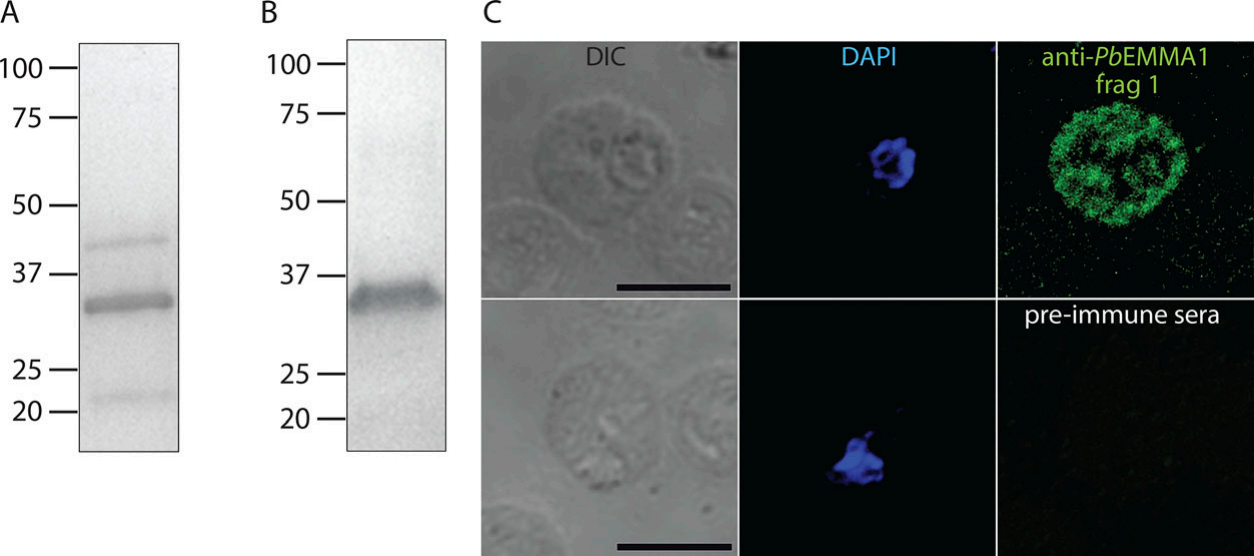
***Pb*EMMA1 studies.(A and B)** Purification of r*Pb*EMMA1 fragments 1 (A) and 2 (B) from *E. coli* inclusion bodies was achieved with sequential fast protein liquid chromatography using nickel chelate affinity chromatography and anion exchange chromatography. Proteins were resolved by SDS-PAGE, and amino acid sequences of all visible bands were verified as portions of *Pb*EMMA1 by LC-MS/MS. **(C)** Permeabilized *Pb*ANKA-infected erythrocytes harvested from mice were probed with mouse anti-*Pb*EMMA1 fragment 1 antibodies or preimmune sera and counterstained with DAPI for immunofluorescence confocal microscopy assays. Scale bar, 5 µm. DIC, differential interference contrast microscopy.

To evaluate the vaccine potential of EMMA1 in vivo, we designed immunization studies in a malaria mouse model using *Pb*ANKA parasites. *Pb*EMMA1 (PBANKA_0914100) and *Pf*EMMA1 have only 26.5% amino acid identity, and identities of fragment 1 and 2 orthologues are 23.4% and 22.0%, respectively. Like *Pf*EMMA1, *Pb*EMMA1 is not essential to murine parasite growth (Bushell et al., 2017). We expressed and purified r*Pb*EMMA1 fragments 1 and 2 and generated mouse antisera against these recombinant proteins (Fig. S5, A and B). We demonstrated labeling of *Pb*ANKA trophozoites in iRBCs from a BALB/cJ mouse with anti-*Pb*EMMA1 fragment 1 but not fragment 2 antibodies (Fig. S5 C).

**Figure 8.**
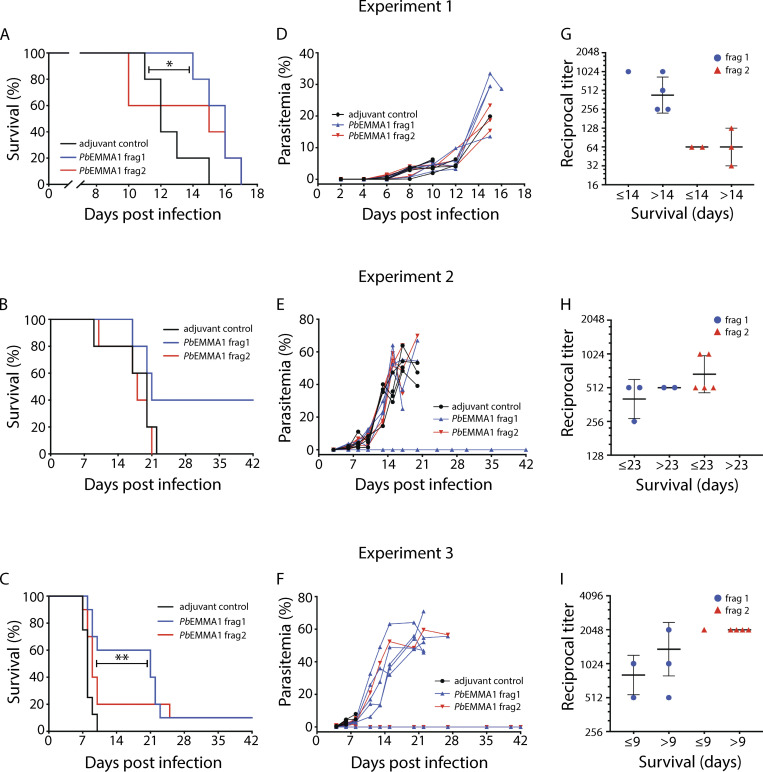
**Survival, parasitemia, and antibody titers in mice immunized with r*Pb*EMMA1 after challenge with *Pb* ANKA.(A)** Experiment 1: BALB/cJ mice immunized three times i.p. with r*Pb*EMMA1 fragment 1 (*n* = 5) survived significantly longer than mice immunized with adjuvant alone (*n* = 5) after i.p. challenge with 10^4^ *Pb*ANKA-infected RBCs. *, P < 0.015. **(B)** Experiment 2: Two of five BALB/cJ mice (40%) immunized four times s.c. with r*Pb*EMMA1 fragment 1 eradicated parasitemia after i.p. challenge with 10^4^ *Pb*ANKA-infected RBCs. **(C)** Experiment 3: BALB/cJ mice immunized four times s.c. with r*Pb*EMMA1 fragment 1 (*n* = 10) survived i.p. challenge with 5 × 10^4^ *Pb*ANKA-infected RBCs significantly longer than mice immunized with r*Pb*EMMA fragment 2 (*n* = 10) or adjuvant alone (*n* = 8). **, P < 0.002. Differences in survival of mice were analyzed using the Kaplan–Meier log-rank test. **(D–F)** Curves representing *Pb* parasite densities for each mouse monitored longitudinally are shown. Mice that survived beyond the first 7 d generally had high parasitemia (>10%). **(G–I)** Antibody endpoint titers were tested 2 wk after the final immunization and before challenge. Titers are expressed as the reciprocal of the serum dilution and are plotted on a log_2_ scale. Anti-*Pb*EMMA1 antibody titers are compared between mice that survived ≤ or > mean + SD of the duration of survival (days) of control mice tested in the same experiment. Distribution of data are shown by geometric mean ± geometric SD. Statistical analyses could not be performed because of small numbers of mice. Frag, fragment.

We evaluated the effectiveness of immunization with r*Pb*EMMA1 to protect BALB/cJ mice against lethal challenge with *Pb*ANKA. We conducted three immunization trials with a total of 58 mice testing different routes of immunization and inoculum sizes. In the first trial (Fig. 8 A), BALB/cJ mice were immunized three times via the i.p. route before i.p. challenge with 10^4^ *Pb*ANKA-infected RBCs. r*Pb*EMMA1 fragment 1–immunized mice survived 1.3 times longer than controls (P < 0.015). However, the Titermax-adjuvanted *Pb*EMMA1 administered i.p. induced chemical peritonitis and debilitated the mice.

We then immunized mice via the s.c. route four times for subsequent experiments. In the second trial (Fig. 8 B), immunized BALB/cJ mice were challenged i.p. with 10^4^ *Pb*ANKA iRBCs. In two of five mice (40%) immunized with r*Pb*EMMA1 fragment 1 (each with baseline antibody titers of 1:512,000 and 2–4 parasites/10,000 RBCs visualized by microscopy on the third day after inoculation), parasites were completely eradicated, and the mice remained healthy for the duration of the study. The remaining three mice had baseline antibody titers <1:512,000 and succumbed within 22 d in a time frame similar to that of control mice.

In the third trial (Fig. 8 C), immunized BALB/cJ mice were challenged i.p. with a fivefold higher inoculum (5 × 10^4^ *Pb*ANKA iRBCs) to test a more rapidly progressive disease model. All mice initially had parasitemia on day 3 documented by microscopy, and all adjuvant control mice died by day 10. The median survival of mice immunized with r*Pb*EMMA1 fragment 1 was 2.6-fold longer than adjuvant controls (21 vs. 8 d, P = 0.005; Fig. 8 C). One mouse (10%) from each group of mice immunized with r*Pb*EMMA1 fragments 1 or 2 (each with antibody titers of 1:2,048,000) eradicated parasitemia by day 4. Notably, the partial protection conferred by active immunization, especially in mice with very high antibody titers, appeared to be independent of the density of circulating parasites (Fig. 8, D–F). The discrepancy in survival of r*Pb*EMMA1 fragment 2–immunized mice between experiments 2 and 3 likely reflects a type II statistical error due to small sample sizes. Antibody titers measured before parasite challenge were not consistently associated with prolonged survival relative to sham-treated mice, but the number of animals was too small for statistical analyses (Fig. 8, G–I).

### Anti-*Pf*EMMA1 antibodies are associated with lower parasitemia in Tanzanian children

**Figure 9.**
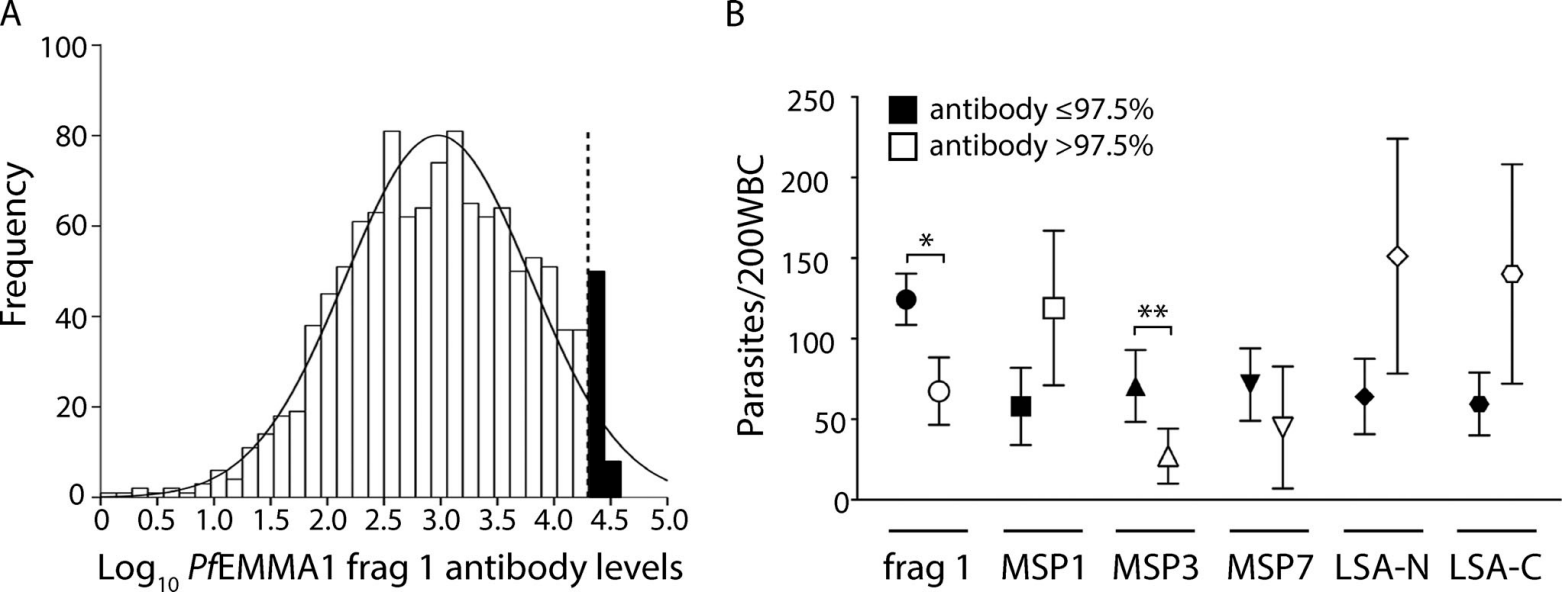
**Antibody levels in Tanzanian children.(A)** The figure shows the overall frequency distribution of log-transformed naturally acquired anti-*Pf*EMMA1 fragment 1 antibody concentrations measured approximately every 6 mo and at sick visits. The curve has a normal distribution; the dashed line denotes the 97.5th percentile, the only threshold among several tested that predicted lower parasite densities. **(B)** The plots show the relationship between antibodies to *Pf*EMMA1 fragment 1 (frag 1), *Pf*MSP1, *Pf*MSP3, *Pf*MSP7, *Pf*LSA-N, and *Pf*LSA-C and parasite densities in Tanzanian children. We measured IgG antibody levels to *Pf*MSP1 (19-kD region of the 3D7 strain; BEI Resources/MR4), *Pf*MSP3 (aa 99–265), *Pf*MSP7 (aa 117–248), *Pf*LSA-N (aa 28–150), and *Pf*LSA-C (aa 1,630–1,909) using available plasma from 225 2-yr-old children enrolled in the Tanzanian birth cohort. The frequencies of contacts with children who had antibody levels ≤97.5th percentile vs. >97.5th percentile were as follows: *Pf*EMMA1 fragment 1, 14,076 vs. 646; *Pf*MSP1-19, 3,186 vs. 95; *Pf*MSP3, 3,202 vs. 79; *Pf*MSP7, 3,183 vs. 98; *Pf*LSA-N, 3,181 vs. 100; and *Pf*LSA-C, 3,203 vs. 78. RRs for these proteins are as follows: *Pf*EMMA1 fragment 1, 0.54 (95% CI, 0.30–0.97, *, P = 0.038); *Pf*MSP1-19, 2.04 (0.92–4.51, P = 0.08), *Pf*MSP3, 0.38 (0.15–1.00, **, P = 0.05); *Pf*MSP7, 0.63 (0.14–2.79, P = 0.54); *Pf*LSA-N, 2.36 (1.05–5.31, P = 0.04); and *Pf*LSA-C, 2.36 (1.15–4.81, P = 0.02). All analyses used multivariable GEE modeling.

To evaluate the impact of naturally acquired antibodies to *Pf*EMMA1 on parasite levels in a human population, we examined children aged 48 wk to 3.5 yr enrolled in a Tanzanian birth cohort (Gonçalves et al., 2014). We measured anti-*Pf*EMMA1 fragment 1 and 2 IgG levels and related these to subsequent parasitemia using multivariable generalized estimating equation (GEE) modeling. This method takes into account within-subject correlations between repeated observations, permits use of all available data, and further adjusts for potential confounding variables (Liang and Zeger, 1986). In total, we obtained 1,274 antibody measurements in plasma from 540 children at scheduled and sick visits (Fig. 9 A). The average time interval between each antibody measurement and either a subsequent antibody determination or completion of the study was 28.1 wk. Subjects were followed for a combined 32,064 child-wk of observation with a total of 14,722 blood smears. Using several defined thresholds of antibody levels to dichotomize the data, only concentrations >97.5th percentile predicted protection against parasitemia. Data were obtained from 51 children at 646 scheduled or sick visits when their anti-*Pf*EMMA1 fragment 1 levels were >97.5th percentile, and from 535 children at 14,076 visits during periods when their antibody levels were ≤97.5th percentile. The distribution of children with antibody levels >97.5th percentile by age was 48 wk (*n* = 6); 76 wk (*n* = 18), 100 wk (*n* = 16), 124 wk (*n* = 8), and 148 wk (*n* = 3).

In our GEE analysis, children with anti-*Pf*EMMA1 fragment 1 antibody levels >97.5th percentile had a 46% reduction in parasite density (Fig. 9 B) after adjustment for potential confounders compared with those with lower antibody levels (rate ratio [RR] = 0.54, 95% CI 0.30–0.97, P = 0.038). Potential confounders included in the model, sickle cell trait (RR = 0.54; 95% CI 0.37–0.77, P < 0.001) and bed net usage (RR = 0.53; 0.38–0.75, P < 0.001), had similar effect sizes as previously reported in this cohort (Gonçalves et al., 2014). We did not detect an association with parasite density for anti-*Pf*EMMA1 fragment 2 antibodies. For comparison, antibody responses to *Pf*MSP3 (RR = 0.38, 95% CI 0.15–1.0, P = 0.05), an established vaccine candidate (Druilhe et al., 2005), but not other recombinant *Pf* proteins (*Pf*MSP1, *Pf*MSP7, or *Pf*LSA), predicted resistance to parasitemia using the same antibody cutoff (97.5th percentile), statistical methods, and dichotomized outcome as for *Pf*EMMA1 (Fig. 9 B). We were not able to detect a significant protective effect of high anti-*Pf*EMMA1 antibody levels against clinically defined severe malaria. However, the small numbers of children with high antibody levels (>97.5th percentile; *n* = 51) and severe malaria (*n* = 53) limited the power of this analysis.

## Discussion

We previously identified the protein encoded by *PF3D7_1134300* as a target of antibodies in plasma from resistant, but not susceptible, Tanzanian children (Raj et al., 2014). Here we demonstrate that the protein is expressed in blood-stage parasites and is dually localized to the RBC and merozoite surfaces. While *Pf*EMMA1 appears to be dispensable for parasite growth in vitro, anti-*Pf*EMMA1 antibodies (murine- and human-derived) specifically inhibit parasite growth in vitro, and *Pf*EMMA1 expression is required for mediating this effect.

We demonstrated that *Pf*EMMA1 sequences from field isolates have substantially less polymorphism compared with other surface antigens in *Pf* (Chan et al., 2014; Wahlgren et al., 2017). This observation suggests there may be a selective constraint preventing the gene sequence from diversifying. Restricted sequence diversity in *Plasmodium* surface proteins has also been observed in *Pf*RH5, a merozoite surface protein, and other newly described single-copy proteins (*Pf*PIESP2 and *Pf*J23) exposed on the RBC surface (Reddy et al., 2015; Nilsson Bark et al., 2018). *Pf*EMMA1 may have limited sequence variation due to (a) restricted display on the surface, (b) limited immune accessibility, (c) immune diversion induced by other surface protein decoys, or (d) *Pf*EMMA1’s tandem repeats, which may mediate evasion of immunity by diverting effective responses away from critical epitopes (Davies et al., 2017; Verra and Hughes, 1999).

*Pf*EMMA1 clusters on some electron-dense knobs in iRBCs, as observed in representative transmission electron microscopy images at a single focal plane, which is consistent with the irregular stippled staining pattern seen in the periphery and on the surface of iRBCs in deconvoluted images of z-stacked multiple planes acquired with confocal fluorescence microscopy. The surface topology may infer a functional role for *Pf*EMMA1 in relation to cytoadhesion and/or immune evasion (Almukadi et al., 2019; Chan et al., 2014; Nacer et al., 2015; Wright and Rayner, 2014). Furthermore, the localization to both RBC and merozoite surfaces may suggest a potential dual function for *Pf*EMMA1 analogous to *Pf*RhopH3, another blood-stage malaria protein, which localizes to both rhoptries in merozoites and plasmodial surface anion channels in iRBCs, where it mediates RBC invasion and nutrient acquisition, respectively (Ito et al., 2017). Multigene STEVOR and SURFIN families are also localized to both RBC and merozoite surfaces, but unlike *Pf*EMMA1, they are highly polymorphic (Khattab and Meri, 2011; Niang et al., 2014; Winter et al., 2005). The exposure of *Pf*EMMA1 on merozoite surfaces presumably explains the high-level GIA mediated by vaccine-induced murine antibodies demonstrated in this report, which is similar to GIA for *Pf*RH5, the leading blood-stage vaccine candidate, based on antibodies derived from rabbits (Douglas et al., 2011) and nonhuman primates (Douglas et al., 2015).

*Pb* mouse models recapitulate various aspects of severe malaria in humans and have been used to test protective immunity of some malaria immunogens (Goodman et al., 2013; Wang et al., 2006). We tested the protective efficacy of *Pb*EMMA1 protein-in-adjuvant immunizations in BALB-cJ mice against *Pb*ANKA in three independent trials. Whereas *Pb*ANKA causes universally fatal disease in mice (Goodman et al., 2013), our findings demonstrated an unprecedented early clearance of parasites in ≤40% of mice or ≤2.6-fold longer median survival (P = 0.005) in lethally infected mice immunized with *Pb*EMMA1 fragment 1 despite very high parasite densities. In addition to humoral immune effectors directed at *Pb*EMMA1 expressed on the RBC surface that limit parasite growth, we speculate that specific antibodies might disrupt parasite protein–endothelial interaction or cellular immune responses that ameliorate disease severity. Further studies are needed to investigate if this protein performs a functional role in the pathogenesis of severe malaria despite being dispensable in blood-stage asexual growth. Although these findings may not be directly translatable to humans because the orthology between *Pf* and *Pb*EMMA1 is very low and *Pb* has a 10–70-fold greater propensity to invade reticulocytes compared with *Pf* (Beeson et al., 2019; Cromer et al., 2006), the rodent model may provide relevant insights into mechanisms and mediators of host defense and immunity (Beeson et al., 2019).

To investigate the potential impact of anti-*Pf*EMMA1 antibodies on malaria in humans, we analyzed a longitudinal cohort of Tanzanian children <3.5 yr of age who had acquired natural anti-*Pf*EMMA1 antibodies. We observed an almost 50% reduction in parasite density among children with anti-*Pf*EMMA1 fragment 1 antibody concentrations >97.5th percentile, which is similar to the degree of protection afforded by sickle cell trait and bed net use in the same cohort. This requirement for very high antibody levels is consistent with the notion that typical ranges of naturally acquired anti-malarial antibodies may not be protective, and all subunit vaccines typically need to induce high levels of antibodies that exceed the expected range of natural antibodies against specific antigens (Ewer et al., 2015; Osier et al., 2014). Using the same antibody cutoffs (97.5th percentile) and statistical methods, we found that antibodies to *Pf*MSP3, a malaria vaccine candidate, were also associated with substantially reduced parasitemia (Druilhe et al., 2005), supporting the validity of our findings.

To prove a causative relationship between anti-*Pf*EMMA1 antibodies and reduced parasitemia, controlled nonhuman primate and human studies will be needed. Further investigation of critical *Pf*EMMA1 epitopes, the role of Ig isotypes/subclasses, complement activation and activation of Fc receptor functions such as opsonic phagocytosis, and antibody-dependent cellular cytotoxicity is needed (Arora et al., 2018; Beeson et al., 2019).

**Figure 10.**
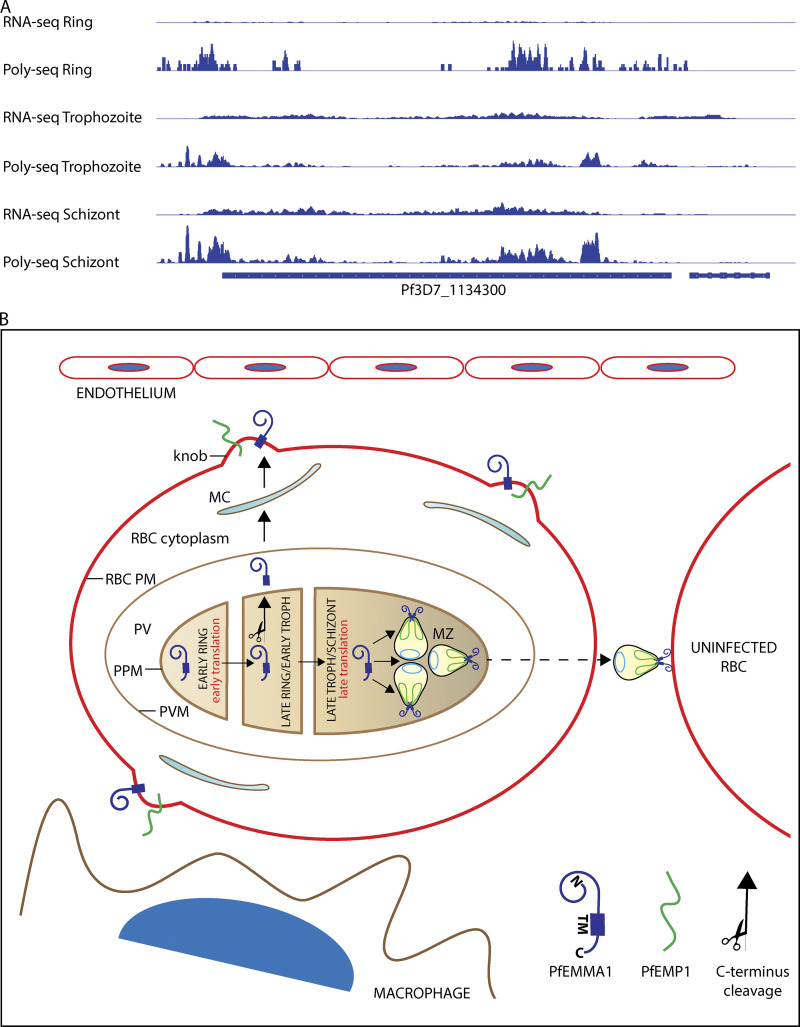
**Expression of *Pf*EMMA1 and proposed export model. (A)** Expression profiles of *Pf*EMMA1. Normalized sequence coverage profiles of steady-state mRNA (RNA-seq) and polysomal mRNA (poly-seq) are shown for *PfEMMA1*. Raw reads were normalized per million mapped reads (Reddy et al., 2015). High coverage of steady-state mRNA is observed at the trophozoite and schizont stages (18 and 36 h, respectively), while coverage of polysomal mRNA is detected at a higher level at the schizont and ring (0 h) stages. **(B)** Proposed compartmentalized model for *Pf*EMMA1 export and localization. Translation of *Pf*EMMA1 is initiated at the ring stage. The C-terminal region is cleaved off, and the truncated protein is exported during the late-ring/early-trophozoite stages via MC to knobs on the RBC exofacial surface with the N-terminus oriented outward. Subsequently, *Pf*EMMA1 is translated in schizonts, and full-length protein is incorporated into merozoites predominantly at the apical surface. C, C-terminus; MZ, merozoite; N, N-terminus; *Pf*EMP1, *Pf* erythrocyte membrane protein 1; PM, plasma membrane; PPM, parasite plasma membrane; PVM, PV membrane; TM, transmembrane domain.

Finally, we propose a theoretical model that unifies our findings and existing RNA sequencing and polysome profiling data to explain the unusual dual-surface localization of this protein (Fig. 1; Bunnik et al., 2013). Similar to *Pf*CLAG9, which is synthesized during two erythrocytic stages (Goel et al., 2010), *PfEMMA1* is transcribed sequentially during the trophozoite and schizont stages, and transcript appears to be retained in merozoites until after reinvasion (Fig. 10 A), which also characterizes other proteins that are exported to the RBC surface (Bunnik et al., 2013; Caro et al., 2014). According to polysome profiling, retained transcript undergoes translation in early ring-stage parasites (Fig. 10 A). Consistent with this timing is the detection of a single protein band in ring-stage parasites (Fig. 1 F). A double protein band is detected in mixed trophozoite/schizont stages when subsequent translation proceeds (Fig. 1 F), which suggests there are two forms of *Pf*EMMA1 (Fig. 10 B): (1) full-length protein containing the C-terminus region that is retained within the parasite (Fig. 5, I and K) and is incorporated into merozoites (Fig. 5, A–H), and (2) a truncated protein, which we hypothesize results from cleavage of the C-terminus region at or near the transmembrane domain. This hypothesis is supported by the fact that the size of an observed small protein fragment labeled with anti–C-terminus antiserum (11.5 kD; Fig. S3 B) approximates the size difference between the two large protein bands labeled with anti-*Pf*EMMA1 fragment 2 antiserum (13 kD; Fig. 1 F), as well as the calculated size of C-terminus region (10.1 kD). The truncated protein appears to be exported and localizes to the RBC exofacial surface (Figs. 2–4). Furthermore, *PfEMMA1* has no introns or identifiable evidence of alternative splicing to explain two differently sized proteins. The precise export signal for this protein is not known, but the hydrophobic α-helical transmembrane domain may mediate protein export as for other PNEPs (Marti and Spielmann, 2013; Spielmann and Gilberger, 2010).

### Conclusion

*Pf*EMMA1 is a novel, conserved blood-stage malaria protein with expression on the surface of both iRBCs and merozoites. Therefore, antibodies to *Pf*EMMA1 could potentially target two sites of vulnerability separated by time and stage of parasite development. Future studies will be needed to delineate the precise functions of *Pf*EMMA1, including its potential roles in RBC invasion, cytoadhesion, and immune regulation. Immunological inhibition of *Pf*EMMA1 could offer new therapeutic options against the most dangerous species causing human malaria.

## Materials and methods

### Reagents

All reagents were obtained from Sigma-Aldrich Corp. unless stated otherwise.

### Parasite population genetics

Genome-wide Variant Call Format files containing variant calls for *Pf* samples collected in Senegal and Malawi were downloaded from the Pf3k project (release 5; https://www.malariagen.net/projects/pf3k; Manske et al., 2012). Analyses were limited to SNPs that fell within coding regions of *PfEMMA1* and other blood-stage vaccine candidates, passed all Pf3K filters, including the GATK VQSLOD filter, and contained only single-clone lineages of *Pf*. Variants were annotated as synonymous or nonsynonymous using the provided SnpEff calls. To identify single-clone infections, low-quality genes (≥25% heterozygous calls or missing calls) were first masked. Then, samples with genome-wide variant sites containing ≥2% heterozygous calls or ≥4% missing calls were removed from further analysis. Genes with either ≥20% heterozygous calls or missing calls in the remaining samples were then masked. Within each sample, any remaining heterozygous calls were transformed into homozygous calls by retaining the allele with highest read support (Early et al., 2018). This filtering provided a set of 99 Senegal and 110 Malawi samples. For each gene, custom Perl scripts were used to calculate pairwise nucleotide diversity (π), Tajima’s *D*, and Weir and Cockerham’s estimate of *F_ST_* (Weir and Cockerham, 1984). The nucleotide diversity metric (π) takes into account the length of the gene, the number of variants, and their frequency in the population. To exclude genes with poor coverage, downstream analyses only included genes that included at least five SNPs.

### Recombinant *Pf* protein expression and purification

We were unable to express the entire *Pf*EMMA1 protein segment identified by our original screen of the *Pf*3D7 cDNA Lambda Zap library (Raj et al., 2014). Therefore, we elected to express two overlapping fragments within that region: *Pf* EMMA1 fragments 1 and 2, which overlap by 38 aa. Codon-optimized *Pf*EMMA1 open reading frames encoding aa 1,164–1,401 (*Pf* fragment 1), aa 1,364–1,600 (*Pf* fragment 2), aa 2,140–2,223 (C-terminus), and *Pb*EMMA1 open reading frames encoding aa 1,141–1,365 (*Pb* fragment 1) and aa 1,343–1,575 (*Pb* fragment 2) were cloned into pD451-SR, an *Escherichia coli* expression vector with an IPTG-inducible T7 promoter and strong ribosome binding site (ATUM), except for *Pf* fragment 2 that was cloned into pET30 (Novagen, EMD Millipore). The plasmids encode a fused S⋅tag on the amino side and 10xHis tags (or 6xHis tags in pET30) on the C-terminal side, to facilitate identification and metal chelate chromatography, respectively. The resulting plasmids were transformed into *E. coli* BL21(DE3) (Novagen, EMD Millipore). Transformants were cultivated in 8-liter batches of Terrific broth with 100 µg/ml kanamycin as previously described (Raj et al., 2014). 50 g of cell paste was resuspended in PBS, 1% Triton X-100, and 100 mM phenylmethylsulfonyl fluoride. Cells were lysed by high-pressure disruption at 20,000 PSI (model 110-T; Microfluidics), and the lysate was then incubated with NP-40 at 4°C for 30 min. Inclusion bodies contained in the pellet were resuspended in PBS using a tissue homogenizer and disrupted under high pressure as before. The resulting pellet was dissolved in buffer containing 8 M urea, 10 mM potassium phosphate, 300 mM NaCl, and 10 mM imidazole.

Recombinant proteins were purified under endotoxin-free conditions using a two-step process on BioPilot chromatography equipment (Pharmacia). First, the dissolved pellet was applied to an AP-1 column (Waters) containing 12 ml of Nuvia IMAC nickel-charged resin (Bio-Rad), and protein was refolded on-column by exchanging buffer containing urea with urea-free buffer over 10 column volumes. Bound protein was eluted with a stepped gradient containing increasing concentrations of imidazole. The fractions containing the protein of interest were pooled and then further purified by anion exchange chromatography using an UNO Q12 column (Bio-Rad). Proteins were eluted with a linear gradient of elution buffer (1 M NaCl, 10 mmol/liter Tris, and 1 mmol/liter EDTA, pH 8.0), and buffer was exchanged into 50 mM sodium phosphate for storage at −80°C, under which conditions proteins were stable for at least 1 yr. The identities of the recombinant polypeptides were confirmed by immunoblots and liquid chromatography tandem mass spectrometry (LC-MS/MS; W.M. Foundation Biotechnology Resource Laboratory). Protein concentrations were measured with a BCA assay kit (Pierce).

*Pf*MSP1 (19-kD region of 3D7 strain from BEI Resources/MR4), *Pf*MSP3 (aa 99–265), *Pf*MSP7 (aa 117–248), *Pf*LSA-N (aa 28–150), *Pf*LSA-C (aa 1,630–1,909), and *Pf*GARP (aa 410–673) were purified as previously described (Raj et al., 2020; Raj et al., 2014). We expressed and purified a region (aa 490–820) of *Pf*SAS4 (spindle assembly abnormal protein 4) encoded by PF3D7_1458500 with immobilized metal affinity chromatography as for *Pf*EMMA1, followed by size-exclusion chromatography. Proteins used for immunizations contained less endotoxin than the threshold pyrogenic dose of 5 EU/kg measured with a chromogenic LAL endotoxin assay that conforms to Food and Drug Administration standards (ToxinSensor; GenScript).

### Parasite strains and cultivation

*Pf* strains representing sialic acid–independent (*Pf*3D7; MRA-102) and sialic acid–dependent (*Pf*W2; MRA-157 and *Pf*Dd2; MRA-150) RBC invasion pathways, and *Pb*ANKA (MRA-311) were obtained from the Biodefense and Emerging Infections Research Resources Repository, National Institute of Allergy and Infectious Diseases, National Institutes of Health, Bethesda, MD. *Pf* strains were cultivated in vitro as previously described (Raj et al., 2014; Trager and Jensen, 1976). Blood smears were prepared and culture medium was exchanged every 48 h.

### Anti-*Pf*EMMA1 Igs

Mouse anti-*Pf*EMMA1 antisera were generated by immunizing BALB/cJ (JAX) mice with 50 µg of r*Pf*EMMA1 fragments emulsified with equal volumes of TiterMax Gold adjuvant (CytRx Corp.) s.c. at 2-wk intervals for four doses. Immune sera as well as BALB/cJ preimmune sera used for control experiments were preadsorbed with fresh uninfected human O^+^ RBCs in a 20:1 volume ratio for 1 h to remove any cross-reactive anti-human RBC Igs, and heat-inactivated at 56°C for 30 min to remove complement. Total IgG and IgM were purified using sequential precipitation with caprylic acid and ammonium sulfate as previously described (Bergmann-Leitner et al., 2008; Perosa et al., 1990) to avoid potential contamination by sodium azide present in small amounts in protein G binding buffer (an alternative method for IgG purification), and the sample was dialyzed against RPMI 1640 (Gibco) in spin columns. Igs were resolved by SDS-PAGE, and identities of IgG and IgM were verified by LC-MS/MS.

To affinity-purify specific human anti-*Pf*EMMA1 antibodies, we coupled 3 mg of r*Pf*EMMA1 to 1 ml of NHS-activated Sepharose 4 Fast Flow chromatography resin (GE Health Sciences) according to the manufacturer’s instructions. Coupled resin was incubated with 3 ml of pooled human plasma (preadsorbed with uninfected human O^+^ RBCs) that we obtained from healthy, HIV-negative, nonpregnant Kenyan adults who were not receiving antimalarial therapy and who participated in a research study in Bondo, Western Kenya, as previously described (Frosch et al., 2017). After extensive washing in PBS and 0.05% Tween 20, bound antibody was eluted in 0.1 M glycine, pH 2.3, and immediately neutralized with 1 M Tris HCl, pH 9.0. Eluted antibodies were dialyzed against RPMI 1640 in spin columns (Amicon Ultra-15; EMD Millipore) and sterilized before use (Ultrafree-MC, 0.22 µm pore; EMD Millipore). Antibody concentrations were measured with a NanoDrop 2000c and confirmed with a BCA protein assay (Thermo Fisher Scientific).

### Polyacrylamide gels and western blots

We prepared lysates of human RBCs that were uninfected or infected with parasites at different stages. SDS-PAGE and western blots were performed as previously described (Raj et al., 2014). Denatured proteins were resolved on either precast 4–15% polyacrylamide Tris-glycine gels or precast 16.5% polyacrylamide Tris-Tricine gels (Bio-Rad) and stained with GelCode Blue Stain Reagent (Thermo Fisher Scientific). Antibodies used for western blots were (a) anti-*Pf*EMMA1 fragment 1 and 2 mouse polyclonal antisera or preimmune sera (1:750) and anti-mouse IgG (Fab specific) conjugated to alkaline phosphatase (Millipore Sigma; 1:5,000), and (b) anti-*Pf*EMMA1 C-terminus mouse polyclonal antisera or preimmune sera (1:200 dilution) and anti-mouse IgG (H&L) conjugated to HRP (Abcam; 1:5,000). *Pf*GAPDH was used as a loading control (mouse anti-*Pf*GAPDH 1:2,000; gift of Claudia Daubenberger, Swiss Tropical and Public Health Institute, Basel, Switzerland). Proteins were visualized using colorimetric or chemiluminescent methods.

### GIA assays

GIAs were conducted with varying concentrations of caprylic acid and ammonium sulfate–purified total Igs as previously described (Malkin et al., 2005) compared with medium and preimmune sera controls. Sorbitol-synchronized trophozoite stage *Pf* parasites (Lambros and Vanderberg, 1979) at ∼0.4% parasitemia and 1% final hematocrit were incubated with sera/Igs in a final volume of 50 µl in microtiter wells for 40 h (one replication cycle). Cultures were performed in triplicate with three biological replicates. Blood films were stained with Giemsa, and microscopists who were blinded to treatment conditions enumerated trophozoite-stage iRBCs among ≥2,000 RBCs per slide. EC_50_ was calculated using nonlinear regression. To test the effect of neutralization of antibodies on growth inhibition, anti-fragment 1 and 2 Igs (2.5 mg/ml each) were preincubated with recombinant fragment 1 and 2 proteins, respectively (650 nM each) at room temperature for 1 h.

### IF assays

Blood smears of asynchronous *Pf*3D7-strain parasite cultures were permeabilized and fixed with 100% methanol at −20°C for 15 min and blocked with PBS/2% BSA. Permeabilized iRBCs were probed with mouse anti-*Pf*EMMA1 antisera (1:250), mouse preimmune sera (1:250), rabbit anti-*Pf*AMA1 or anti-*Pf*RH5 (1:500; gifts of S.J. Draper, University of Oxford, Oxford, England), rabbit anti-*Pf*REX1 or -*Pf*SBP1 (1:2,000 and 1:5,000, respectively; gifts of T. Spielmann, Bernhard Nocht Institute for Tropical Medicine, Hamburg, Germany), rabbit-anti-GPA or -GPC (1:500; Abcam), and rabbit anti-*Pf*MSP1 or -4 (1:500; BEI Resources/MR4). Blood smears were incubated with primary antibodies for 2 h at 25°C, washed in PBS, and incubated with goat anti-mouse IgG conjugated with Alexa Fluor 488 (Invitrogen) and goat anti-rabbit IgG conjugated with Alexa Fluor 594 (Invitrogen) both at 1:2,000 for 1 h at 25°C. After washing with PBS, iRBCs were mounted onto glass slides with Vectashield Antifade Mounting Medium (Vector Laboratories) containing DAPI to label nuclei.

To evaluate surface localization in live, nonpermeabilized iRBCs, we performed live-cell staining on sorbitol-synchronized late-stage *Pf*3D7-infected RBCs that were enriched with LS columns and a QuadroMACS separator (Miltenyi Biotec). 10^8^ live iRBCs were blocked for 1 h at 4°C in PBS/2% BSA. Nonpermeabilized iRBCs were incubated with anti-*Pf*EMMA1 mouse antisera (1:15) or preimmune sera (1:15) and anti-*Pf*MSP4 (1:15; BEI Resources/MR4) or anti-GPC rabbit polyclonal antibodies (1:15; Abcam) in PBS/2% BSA for 2 h at 4°C. After washing with PBS, samples were incubated with secondary antibodies at 4°C as described above. Washed cells were resuspended in PBS, and blood smears were fixed with 100% methanol for 15 min at −20°C. Slides were covered with Vectashield (Vector Laboratories) antifade mounting medium containing DAPI to label nuclei.

Slides were imaged with a Nikon C1si confocal microscope using diode lasers at 402, 488, and 561 nm. Serial optical sections were obtained sequentially with EZ-C1 computer software’s frame lambda, which is frame sequential scanning. Frame lambda reduces potential bleed-through when emission spectra overlap. The images of iRBCs were collected at 0.1 μm with a 100× Plan Apo lens with a 1.4 numerical aperture and a scan zoom of 4. In each experiment, acquisition settings were determined by the most brightly stained slide. All subsequent slides were collected with the same parameters. Deconvolution image processing was performed with Nikon’s Elements software v3.2 before colocalization analysis and optimization in Adobe Photoshop. All adjustments made in Photoshop were optimized in the brightest image, and the same changes were applied to all images within an experiment. For each colocalization study, 10 regions of interest were outlined, and each Z stack was analyzed for Pearson’s correlation. A coefficient factor >0.5 was considered indicative of colocalization.

### Immuno-transmission electron microscopy

Mixed trophozoite- and schizont-stage *Pf*3D7-infected human O^+^ RBCs were enriched with LS columns and a QuadroMACS separator (Miltenyi Biotec) to >80% purity by light microscopy. Aliquots of 10^8^ live iRBCs were blocked for 1 h at 4°C in 1× PBS containing 2% BSA. A sample of iRBCs was permeabilized by treatment with 0.1 µg EqtII (gift of G. Anderluh, National Institute of Chemistry, Ljubljana, Slovenia) for 6 min at 25°C (Jackson et al., 2007) and then washed with PBS. EqtII is a pore-forming toxin that lyses RBC membranes but not MC or PV membranes, releases cytoplasmic contents including hemoglobin, and permits penetration of antibodies for immunogold labeling. Samples of permeabilized or intact iRBCs were incubated with anti-*Pf*EMMA1 fragment 2 mouse antisera (1:15) or preimmune mouse sera (1:15) and anti-GPC rabbit polyclonal antibodies (1:15; Abcam) in PBS and 0.1% BSA-c (Aurion) for 1 h at 4°C. After washing with PBS, iRBCs were incubated with 6 nm gold-conjugated anti-mouse IgG and 10 nm gold-conjugated anti-rabbit IgG (Aurion) in PBS and 0.1% BSA-c for 1 h at 4°C. Washed cell pellets were layered with 2% glutaraldehyde and 1% paraformaldehyde in 0.1 M sodium cacodylate buffer and allowed to fix for 1 wk at 4°C. Cell pellets were postfixed for 40 min with 0.5% osmium tetroxide, buffer rinsed, dehydrated in a graded acetone series, infiltrated, and embedded in Spurr’s epoxy resin. The embedded pellets were cut from the resin-filled microfuge tubes and attached to blank epoxy blocks. Ultrathin sections (50–60 nm) were retrieved onto 300-mesh copper grids and contrasted with uranyl acetate. Sections were examined at 80 kV using a CM-10 electron microscope (FEI). Images were obtained with a model 785 Erlangshen ES1000W charge-coupled device camera (Gatan).

### *Pf*EMMA1 gene KO

Primer pairs are listed in Table S1. A *PfEMMA1* gene KO plasmid was constructed by amplifying 5′ (443 bp) and 3′ (582 bp) regions of the gene using primer pairs cfp147 + cfp148 and cfp149 + cfp150, respectively, and along with two guide RNAs that recognize PAM sequences at the 5′- and 3′- end of the gene, were cloned into a modified pUF1-2xT7 vector that contains two T7 cassettes for expression of two guide RNAs. The donor vector was designed so that successful chromosomal editing would result in the deletion of 5,601 bp of the gene and insertion of the yeast *dihydroorotate dehydrogenase* (*DHODH*) gene, with *Pf*Cam 5′ and *Pf*Hrp2 3′ regulatory sequences, as a positive selectable marker for parasite transfections (Wagner et al., 2014). The donor vector was sequence verified, and inserts were further confirmed by restriction digestion. *Pf*EMMA1_pUf1-2xT7 was electroporated into *Pf*NF54^Cas9+T7 polymerase^ parasites (Nasamu et al., 2021) as described previously (Deitsch et al., 2001). Briefly, 50 µg of purified plasmid DNA was mixed with human RBCs and subjected to 8 square wave electroporation pulses of 365 V for 1 ms each, separated by 0.1 s, in a 0.2-cm cuvette. Drug selection with 1.5 µM DSM1 (Calbiochem) was initiated 4 d after parasite addition, and the emergence of transfectants was monitored with Giemsa-stained smears. *Pf*EMMA1_pUf1-2xT7 was transfected in triplicate. After parasites came up, clonal parasites were obtained by limiting dilution (Rosario, 1981). PCR was performed on gDNA extracted from transfected parasites to validate integration at the 5′ region of the locus using primers cfp242 (P1), cfp243 (P2), and cfp244 (P3) and the 3′ region of the locus using primers cfp245 (P6), cfp246 (P5), and cfp247 (P4). We used NF54^attB^ containing the human *dhfr*-positive selectable marker as a control parasite, which we selected with WR99210 (Adjalley et al., 2011).

### Mouse immunization regimens and antibody assays

Groups of 6–8-wk-old female BALB/cJ mice were immunized twice weekly with 50 µg recombinant proteins and an equal volume of TiterMax Gold (CytRx Corp.) via three i.p. or four s.c. injections. Antibody assays were performed with r*Pb*EMMA1 fragment 1– and 2–coated Bio-Plex COOH beads (Bio-Rad) as previously described (Raj et al., 2014). The endpoint titer of serially diluted mouse sera was determined 2 wk after final immunization before parasite challenge to determine antibody concentrations. Mice were challenged with 10^4^ or 5 × 10^4^ *Pb*ANKA-infected RBCs and were monitored with blood films daily from day 2 to 5 after challenge and then on alternate days to quantify parasitemia. Mice exhibiting signs of cerebral malaria (seizure or paresis) or excessive weight loss were euthanized in accordance with the approved animal protocol.

### Statistics

For immunoepidemiologic analyses, subjects were enrolled in the Mother Offspring Malaria Studies (MOMS) project, which was based at Muheza Designated District Hospital in northeastern Tanzania. Details of the MOMS study design, enrollment, methods, case definition of disease severity, and exclusion criteria have been published previously (Raj et al., 2014). A multivariable GEE model with a ɣ distribution (Liang and Zeger, 1986) was used to assess the impact of anti-*Pf*EMMA1 antibody concentrations (exposure) on malaria parasite burden and clinical severity (outcomes) in Tanzanian children from 48 wk to 3.5 yr of age. Children in the original sample used for the *Pf*3D7 cDNA phage screen were excluded from these analyses (Raj et al., 2014). Blood for antibody testing was obtained at scheduled visits approximately every 6 mo. Antibody concentrations were measured with r*Pf*EMMA1 fragment 1– and 2–coated Bio-Plex COOH fluorescent beads (Bio-Rad). Blood smears and clinical assessments were scheduled every 4 wk and were also performed at unscheduled sick visits (village-health worker visits, walk-in visits, and hospitalizations). GEE was selected because it takes into account within-subject correlations between repeated observations in a longitudinal study comprising all available data regardless of normality (Liang and Zeger, 1986). The outcome (parasite density) for each subject was analyzed after measuring an antibody level and before the subsequent antibody test derived from all points of contact in an iterative process, using the last observation carried forward method. To determine if any particular threshold could predict outcomes, antibody concentrations were dichotomized at several cutoffs (50th, 75th, 90th, and 97.5th percentiles) and tested simultaneously. We selected the robust covariance matrix estimator and exchangeable correlation structure, which provided the lowest Quasilikelihood Information Criteria level for the full model; the analysis with first-order autoregressive, m-dependent, independent, and unstructured correlation structures produced similar results. In addition to assessing the main effects of anti-*Pf*EMMA1 fragment 1 and 2 antibody levels, several potential confounders were evaluated including age, birth weight, bed net usage, hemoglobin concentration, hemoglobin phenotype, parity, placental malaria, prematurity, scheduled or sick visit, and season at time of birth. A quadratic term for age (age^2^) was also included in the model because there appeared to be a curvilinear relationship between age and parasitemia.

Differences in survival of groups of mice were analyzed using the Kaplan–Meier log-rank test. Differences in parasitemia density assessed by GIA were analyzed using Student’s *t* test. A two-tailed P < 0.05 was considered to be statistically significant. Statistical analyses were performed with SAS 9.3, SPSS 24.0, and Prism 7 software.

### *Pf* expression profiling

Sources of data and methods for analyzing steady-state mRNA and polysomal mRNA levels for *Pf*EMMA1 in the intraerythrocytic developmental cycle have been described previously in detail (Bunnik et al., 2013).

### Study approval

Rhode Island Hospital’s Institutional Animal Care and Use Committee and Institutional Review Board approved the study. Human samples and data were obtained under protocols approved by the institutional review boards of the Seattle Biomedical Research Institute and the University of Minnesota, the Medical Research Coordinating Committee of the National Institute for Medical Research in Tanzania, and the Ethics Committee at the Kenya Medical Research Institute. Informed consent for use of plasma samples and clinical data were obtained from parents or legal guardians of all participants. Animal experiments were conducted in full compliance with guidelines approved by the Rhode Island Hospital’s Institutional Animal Care and Use Committee.

### Online supplemental material

Fig. S1 shows a low degree of genetic variance for *Pf*EMMA1 between parasite populations in Senegal (West Africa) and Malawi (southeastern Africa) as indicated by the Weir and Cockerham’s estimate of *F_ST_*. Also shown is full-length native *Pf*EMMA1 protein in an immunoblot of *Pf*3D7-infected erythrocytes probed with affinity-purified antibodies from malaria-immune Kenyan adults. Fig. S2 provides additional evidence for the localization of *Pf*EMMA1 by immunofluorescence confocal microscopy using mouse anti-*Pf*EMMA1 fragment 1 antisera. Fig. S3 includes an immunoblot showing r*Pf*EMMA1 C-terminus expressed in *E. coli* and purified with sequential chromatography. Also shown is native *Pf*EMMA1 C-terminus in an immunoblot of *Pf*3D7-infected erythrocytes probed with mouse anti–C-terminus antisera. Fig. S4 provides additional evidence of dose-dependent inhibition of *Pf* W2 growth/invasion by murine anti-*Pf*EMMA1 antisera. Fig. S5 includes an immunoblot showing r*Pb*EMMA1 fragments 1 and 2 expressed in *E. coli* and purified with sequential chromatography. Also shown is a representative *Pb*ANKA-infected mouse erythrocyte that is permeabilized and labeled with murine-anti-*Pb*EMMA1 fragment 1 antisera, indicating the localization of native *Pb*EMMA1. Table S1 lists the oligonucleotides used to delete the native NF54 *PfEMMA1* locus.

## Supplementary Material

Table S1 lists the oligonucleotides used to delete *PfEMMA1* gene.
