# Supplementary material for: A newly characterized malaria antigen on erythrocyte and merozoite surfaces induces parasite inhibitory antibodies
Source: J Exp Med. 2021 Aug 3;218(9):e20200170. doi: 10.1084/jem.20200170 (PMC8340565; doi:10.1084/jem.20200170)
Supplement: Table S1 — lists the oligonucleotides used to delete PfEMMA1 gene. [file JEM_20200170_TableS1.docx]

**Supplemental Table 1.** **List of oligonucleotides used to delete *PfEMMA1* gene.**

| **Name** | **Nucleotide sequence** | **Description** |
| --- | --- | --- |
| cfp147 | aatatatatccaatggcccctttccgcggggaggactagtGTGTTCTTGAATTTCATGGG | PF3D7_1134300_pUF1_2xT7 LHR forward |
| cfp148 | atatattttaattttttttacaaaatgcttaagCTCTCTTCTTTTTTATATTACAAGTATTCA | PF3D7_1134300_pUF1_2xT7 LHR reverse |
| cfp149 | ttatatataagaacatatttattaaatctagaattcGAGAAGACGAACAAGAAATAAAAG | PF3D7_1134300_pUF1_2xT7 RHR forward |
| cfp150 | ctaaacgggtcttgaggggttttttgagttatcgatatgaattcCATTAATACATAAATAGTTACTTCCATCAC | PF3D7_1134300_pUF1_2xT7 RHR reverse |
| cfp151 | aggtgacactatagaatactctaatacgactcactatagGCTAGTACCTTTCATACCAT | PF3D7_1134300_pUF1_2xT7 guide1 forward |
| cfp152 | actagccttattttaacttgctatttctagctctaaaacATGGTATGAAAGGTACTAGCc | PF3D7_1134300_pUF1_2xT7 guide1 reverse |
| cfp153 | gactagccttattttaacttgctatttctagctctaaaacCTTTGCGTTCATTTTTCTCG | PF3D7_1134300_pUF1_2xT7 guide2 forward |
| cfp154 | gcctaggagttcctaggagtttaatacgactcactataggCGAGAAAAATGAACGCAAAG | PF3D7_1134300_pUF1_2xT7 guide2 reverse |
| cfp242 (P1) | ATTTGGCATTATATATTGTAACAATTTGAT | PF3D7_1134300 integration check 5' For |
| cfp243 (P2) | CATCATCATCATCAAAATAAATACCGTTA | PF3D7_1134300 integration check native locus 5' Rev |
| cfp244 (P3) | CATTGAGTTGATACTGCCTAGAG | PF3D7_1134300 integration check edited locus 5' Rev |
| cfp245 (P6) | GTTTACATACATAATGTTTACTTCTGGT | PF3D7_1134300 integration check 3' Rev |
| cfp246 (P5) | TCTTCAGTATCTAACCTAAAAAGC | PF3D7_1134300 integration check native locus 3' For |
| cfp247 (P4) | GCTAAGGGTTATACATCCATAGA | PF3D7_1134300 integration check edited locus 5' For |
